# Supplementary material for: Random allelic expression in the adult human body
Source: Cell Rep. Author manuscript; Available in PMC 2023 Sep 7. (PMC10484211; doi:10.1016/j.celrep.2022.111945)
Supplement: 1 [file NIHMS1870509-supplement-1.pdf]

Cell Reports

Resource

Random allelic expression in the adult human body

Graphical abstract

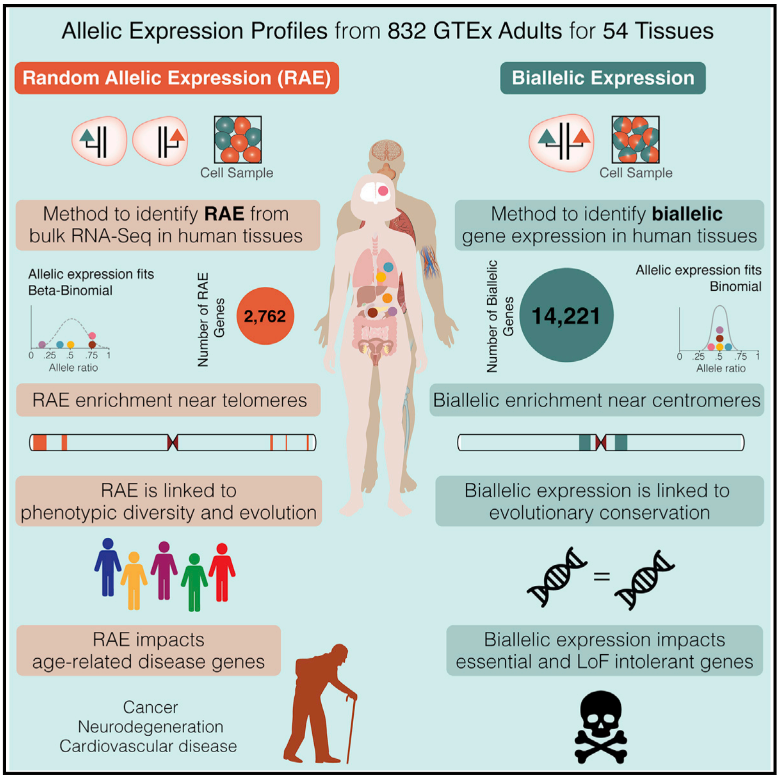

Authors

Stephanie N. Kravitz, Elliott Ferris, Michael I. Love, Alun Thomas, Aaron R. Quinlan, Christopher Gregg

Correspondence

chris.gregg@neuro.utah.edu

In brief

Kravitz et al. develop a method to identify genes that randomly differentially express their parental alleles in human tissues and find that random allelic expression disproportionately impacts genes linked to age-related disease, GWAS hits, and rapidly evolving genomic regions. They also find putative subtypes from different gene regulatory features and domains.

Highlights

- An RNA-seq method to profile random allelic expression (RAE) in human tissues
- Identification of 2,762 high-confidence random allelic genes
- RAE impacts rapidly evolving genes implicated in disease and phenotype variation
- Putative subtypes of RAE are revealed based on gene regulatory features

Kravitz et al., 2023, Cell Reports *42*, 111945
January 31, 2023 © 2022 The Author(s).
https://doi.org/10.1016/j.celrep.2022.111945

CellPress

Cell Reports

CellPress
OPEN ACCESS

Resource

Random allelic expression in the adult human body

Stephanie N. Kravitz,[1,2] Elliott Ferris,[2] Michael I. Love,[4,5] Alun Thomas,[3] Aaron R. Quinlan,[1,6] and Christopher Gregg[1,2,6,7,*]
[1]Department of Human Genetics, University of Utah, Salt Lake City, UT, USA
[2]Neurobiology, University of Utah, Salt Lake City, UT, USA
[3]Department of Internal Medicine, Epidemiology, University of Utah School of Medicine, Salt Lake City, UT, USA
[4]Department of Biostatistics, University of North Carolina at Chapel Hill, Chapel Hill, NC, USA
[5]Department of Genetics, University of North Carolina at Chapel Hill, Chapel Hill, NC, USA
[6]These authors contributed equally
[7]Lead contact
*Correspondence: chris.gregg@neuro.utah.edu
https://doi.org/10.1016/j.celrep.2022.111945

SUMMARY

Genes are typically assumed to express both parental alleles similarly, yet cell lines show random allelic expression (RAE) for many autosomal genes that could shape genetic effects. Thus, understanding RAE in human tissues could improve our understanding of phenotypic variation. Here, we develop a methodology to perform genome-wide profiling of RAE and biallelic expression in GTEx datasets for 832 people and 54 tissues. We report 2,762 autosomal genes with some RAE properties similar to randomly inactivated X-linked genes. We found that RAE is associated with rapidly evolving regions in the human genome, adaptive signaling processes, and genes linked to age-related diseases such as neurodegeneration and cancer. We define putative mechanistic subtypes of RAE distinguished by gene overlaps on sense and antisense DNA strands, aggregation in clusters near telomeres, and increased regulatory complexity and inputs compared with biallelic genes. We provide foundations to study RAE in human phenotypes, evolution, and disease.

INTRODUCTION

Human phenotypes and disease risks are variable for reasons not fully understood. For example, a typical healthy individual carries over 100 putative loss-of-function genetic variants yet can show no overt clinical symptoms.[1] Moreover, the same gene is often implicated in multiple disorders, obfuscating disease etiology.[2–6] Genetic variants affecting human phenotypes and disease risks are typically heterozygous, contributing to a growing interest in understanding interactions between genetic variation and allele-specific expression (ASE).[7] Epigenetic mechanisms are important in regulating ASE, as shown for imprinted genes and genes subject to random X chromosome inactivation (XCI).[8] For a heterozygous variant, preferential expression of a pathogenic parental allele can increase phenotypic effects and disease risks, while the reverse can suppress them. For example, in females with Rett syndrome, caused by mutations in the X-linked gene *MECP2*, XCI skewing of the chromosome carrying the pathogenic allele affects the penetrance of the mutation and severity of the neurodevelopmental phenotype.[9–11] These studies show that ASE influences the phenotypic effects of a genetic variant or mutation, and given these potential impacts it is important to determine the genome-wide landscape of non-genetic allelic effects in human tissues and the identity of genes affected.

Previous studies of human and mouse cell lines found that many autosomal genes (~1%–15%) can undergo random monoallelic expression (aRME) with properties such as random X-inactivation, including clonal inheritance of monoallelic expression states.[12,13] Some findings have been challenged by single-cell RNA sequencing (scRNA-seq) studies, which claim clonal RME (cRME) is very rare (<1%).[14–18] Instead, these studies observed widespread dynamic RME (dRME) whereby ASE states differed between cells in a single clone. dRME was found to impact as many as 85% of genes in T cells. However, these conclusions are also debated,[19] and other scRNA-seq studies have also reached opposing conclusions on the prevalence of any RME.[20,21] Thus, the nature of the different RME effects that exist remains an important area of study. One study analyzing allelic expression for *Bcl11b* in mouse T cell lines directly showed stable, clonal, and functionally important RME that manifests at the protein level to control cell-fate decisions.[22] RME at the protein level was recently described in *C. elegans*[23] and *Drosophila*,[24] and an analysis of select genes in F1 hybrid mouse cell lines validated clonal random monoallelic expression in neural precursor cells.[25] Overall, autosomal RME and other forms of non-canonical allelic regulation are emerging as important modes of gene regulation that could impact development and disease by affecting gene dose, the expression of heterozygous genetic variants and mutations, and cellular molecular diversity.[8] However, most existing evidence comes from cell lines and indirect chromatin signatures.[26,27] Thus, there is a need to investigate these allelic phenomena directly in human tissues and better understand the affected genes, biological pathways, disease processes, mechanisms, and subtypes.

Previously, we devised a genomics and statistical framework to profile and discover non-genetic modes of random allelic expression (RAE) in mouse and primate tissues using bulk tissue RNA-seq.[28] Our bulk RNA-seq approach is designed to help

Check for updates

This is an open access article under the CC BY license (http://creativecommons.org/licenses/by/4.0/).

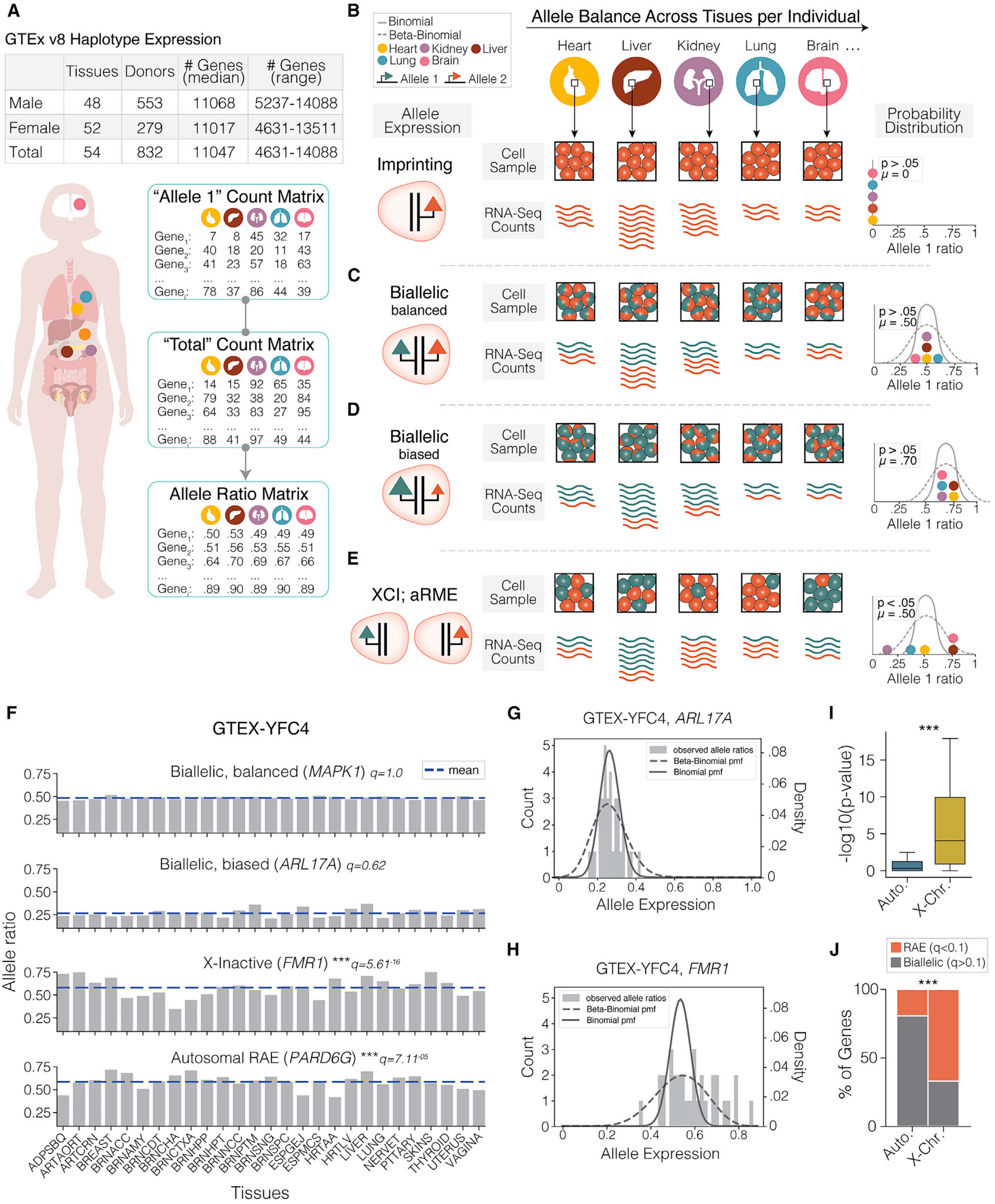

(legend on next page)

CellPress
OPEN ACCESS

overcome the high technical noise suffered by allele-specific scRNA-seq and broadly profile diverse modes of RAE, potentially including cRME, dRME, and other non-genetic random allelic effects. We found RAE in mouse and primate tissues that were validated with multiple orthogonal approaches.[28] Here, we advance this work and develop a methodology to perform genome-wide profiling of RAE in human tissues. We apply our method to >15,000 RNA-seq ASE datasets from 54 human tissues and 832 adults studied by the Genotype Tissue Expression (GTEx) consortium.[29–31] In control studies, our approach robustly differentiates randomly XCI genes from most autosomal genes in female subjects. Moreover, a subset of autosomal genes exhibits RAE characteristics similar to those of X-linked genes. We identify high-confidence genes with RAE (hc-RAE) from a population study of 293 females and 553 males, controlling for expression level, *cis*-eQTLs (expression quantitative trait loci), read mappability, and other confounders. We also identify high-confidence "biallelic genes" (hc-Biallelic) distinguished by robust co-expression of both parental alleles. RAE genes in human tissues are enriched in distinct biological pathways compared with biallelic genes and show significant associations with age-related diseases, including cancer and neurodegenerative processes. We identify putative subtypes of RAE based on associations with different candidate regulatory mechanisms and show that RAE and biallelic genes are under different degrees of genetic constraint. Overall, we uncover high-confidence random allelic genes in adult human tissues and show important links to adaptive cellular processes, age-related diseases, and evolution.

RESULTS

Random allelic expression revealed in human tissues using bulk RNA-seq and whole-genome data

Our starting objective was to identify genes with significant RAE in the tissues of a single person and differentiate those from biallelic genes that co-express both parental alleles. To achieve this, we developed an approach that evaluates a gene's allelic expression variance from haplotype-phased RNA-seq GTEx datasets for multiple different tissues in a single subject[29–31] (Figures 1A and S1A). Because the different tissue samples collected for a single individual are genetically identical, the impact of genetic variation on allelic expression is minimized and enriches for non-genetic effects compared with analyzing samples from different individuals. In this approach, we expect that genes equally co-expressing both parental alleles (biallelic genes) in each cell will show low variance in the relative expression of the two alleles across different tissue RNA-seq datasets from a single person. Thus, the probability of sequencing either allele fits a binomial distribution (Figure 1C). That is, for every gene (i), given allele expression counts (X), and total expression counts (N) for all tissues, the expected probability (p) of sequencing a read for "Allele A" is $p = X_A/N$ and for "Allele B" is $p = X_B/N$. Biallelic expression is the null hypothesis for each gene tested, whereby $H_0{:}X_i \sim \text{binomial}(N_i, p)$ (Figure S1B). Importantly, genes exhibiting biased allelic expression due to genetic *cis*-eQTL or genomic imprinting effects that consistently bias one allele in cells will also fit the binomial model (Figures 1B and 1D). In contrast, genes exhibiting RAE at the cellular level, such as X-linked or autosomal random monoallelic expression (RME), show high allele expression variance across tissues due to clonal or stochastic cellular mosaicism, such that the relative expression of Allele A versus Allele B exhibits overdispersion relative to the binomial (Figure 1E). Consequently, genes with RAE will show a significantly improved fit to the beta-binomial ($H_1{:}X_i \sim \text{beta-binomial}(N_i, \alpha, \beta)$) compared with the binomial (H_0) distribution. Thus, from RNA-seq data collected from multiple tissues, we determine the distribution of allelic expression ratios observed across the tissues and apply a likelihood ratio test to compare the fit of the two distributions in a manner accounting for expression level. This approach reduces false positives for RAE that might be related to low gene expression because low read counts provide insufficient evidence to reject the binomial null hypothesis. Therefore, by analyzing a gene's allelic expression variance across different tissues, our test differentiates significant RAE from biallelic expression or imprinting, enabling genome-wide profiling of RAE in human tissues.

We illustrate our method for one female GTEx individual (GTEX-YFC4) (Figure 1F). For example, the autosomal gene *MAPK1* exhibits little variance in the relative expression of the

Figure 1. An approach to define genes with biallelic and random allelic expression from bulk RNA-seq data

(A) Schematic and table of RNA-seq datasets from GTEx, composed of haplotype-resolved allele-level counts for multiple tissue per individual, with tissue and donor summary data shown in table.

(B) Schematic of different subtypes of allelic expression and the expected allele counts from bulk RNA-seq counts across tissue biopsies. Expected allele counts of an imprinted gene (orange allele expressed) from a cell sample are shown. Allele ratios for each tissue are shown as a histogram with hypothetical binomial and beta-binomial distributions to illustrate how each allelic subtype would fit under either distribution.

(C–E) Schematic of expected allele counts and allele ratio distribution for a (C) biallelic gene with balanced expression of the two alleles, (D) biallelic gene in which one allele is more highly expressed, and (E) X-inactive or autosomal RME gene.

(F) Observed allele ratios across 27 tissue samples from female subject GTEX-YFC4 for four allelic subtypes: biallelic balanced, biallelic imbalanced, X-inactive, and aRME. (MAPK1, q = 1.0; ARL17A, q = 0.62; FMR1, q = 5.61×10^{-16}; PARD6G, q = 7.11×10^{-5}. See supplemental information for full tissue names.

(G) Observed allele ratios (27 tissues) for biallelic gene *ARL17A* for subject GTEX-YFC4, and the binomial and beta-binomial probability mass function distributions for these data. The data fit the binomial null hypothesis (q = 0.62), and therefore exhibit biallelic expression in this individual.

(H) Observed allele ratios (27 tissues) for XCI gene *FMR1* for subject GTEX-YFC4, and the binomial and beta-binomial probability mass function distributions for these data. The data reject the binomial null hypothesis (q = 5.61×10^{-16}) and is better fit by the beta-binomial, and therefore exhibits RAE.

(I) $-\log_{10}$(p value) results for autosomal (n = 12,568) and XCI (n = 109) genes for 34 tissue samples from GTEX-YFC4 (Kruskal-Wallis ANOVA, p = 3.99×10^{-27}). Box plot shows median, interquatile range, and min-max values.

(J) Proportion of significant genes on the autosomes and X chromosome. Benjamini-Hochberg corrected p values (FDR 10%) for GTEX-YFC4 generated using all tissues (Fisher's exact test, p = 5.85×10^{-27}).

*p < 0.01, **p < 0.001, ***p < 0.0001.

two alleles across 39 different brain regions and tissues (27 shown) and is thus scored as biallelic because of the failure to reject the binomial null hypothesis (Figure 1F, *MAPK1*). Additionally, *ARL17A* shows a biased expression of one allele, presumably due to a *cis*-eQTL effect, but maintains consistency in the relative expression of the two alleles across different tissues, and therefore RAE is not detected (Figure 1F, *ARL17A*). In contrast, the randomly inactivated X-linked gene, *FMR1*, displays a high level of variance in allelic expression across different tissues and is correctly identified as having significant RAE based on a significant beta-binomial over binomial fit (Figure 1F, *FMR1*). Finally, *PARD6G* is an example of an autosomal gene with significant RAE and shows allelic variance similar to *FMR1* (Figure 1F, *PARD6G*). The resulting binomial and beta-binomial distributions fit from the allelic expression data for biallelic *ARL17A* (Figure 1G) and random allelic *FMR1* (Figure 1H) further demonstrate how overdispersion is used to identify RAE. These examples illustrate our approach to uncovering significant RAE genes in one person.

We next applied our approach genome-wide to the haplotype-phased allele-specific RNA-seq data for all genes expressed in this female individual. As expected, significantly more XCI genes (Table S1) show RAE compared with autosomal genes (Figure 1I). After correcting for multiple testing, most X-linked genes (66.9%) show RAE and significantly fit the beta-binomial, while most autosomal genes (80.7%) fit the binomial model, consistent with presumed biallelic expression, and the difference in proportions is significant (Figure 1J). Of note, many of the XCI genes not significant for RAE in this individual are likely false negatives due to insufficient power to reject the binomial null hypothesis because they are expressed in significantly fewer tissues than significant XCI genes (mean = 8.77 versus 16.27 tissues; Kruskal-Wallis ANOVA, $p = 1.4 \times 10^{-12}$) and have lower expression (mean = 2.72 transcripts per million [TPM] versus 34.47 TPM; Kruskal-Wallis ANOVA, $p = 2.3 \times 10^{-28}$). These results show that our approach robustly detects RAE for positive control random allelic XCI genes in females, and our analysis uncovered 2,424 significant autosomal genes, revealing putative RAE for ~20% of autosomal genes in this female individual (false discovery rate [FDR] 10%). We next evaluated possible technical confounders and then expanded our analysis to the full GTEx population to define a set of high-confidence, reproducible autosomal RAE genes.

Technical factors are not major contributors to significant RAE gene scores, indicating that our approach predominantly detects biological RAE

Genes with RAE in our study are defined as genes with increased random allelic expression variance across tissues such that the data reject the null hypothesis of biallelic expression following the binomial distribution. As shown above, this approach uncovers a discrete subset of genes, and we tested whether technical confounders that are not related to allelic expression variance are major contributors to false positives with this approach. First, prior to testing for RAE in the GTEx datasets, we removed obvious confounders, including genes with low expression (read depth <10), tissue-specific expression (expressed in <3 tissues per person), and all paralogous and low-mappability genomic regions (Figure S1A). Second, using haplotype-phased reads increases the accuracy of the allelic expression data. Next, we used a regression analysis to determine the impact of a wide range of technical factors that could contribute to false RAE detection in GTEx individuals (gene q values) and the population (gene *Z* scores), including gene expression level, SNP number, eQTL number, mappability, gene length, library depth, tissue number, and more (Figure 2A). We determined the R^2 for each factor as an explanatory variable for individual and population RAE. The results show that none of the technical factors are important contributors to RAE detection in individuals. However, at the population level, gene expression level, SNP number, and tissue number each explained 8%–11% of the variance in the RAE score, raising concerns that low expression, few SNPs (i.e., poor allelic expression resolution), and few tissues (i.e., low sampling) could be technical confounders at the population level. However, we investigated each parameter for the significant RAE genes compared with genes that show biallelic expression and found the opposite. RAE genes detected by our study have increased expression (Figure 2B), increased SNP numbers per kilobase (i.e., improved allelic expression resolution) (Figure 2C), increased numbers of tissues for assessment (Figure 2D), and no difference in mappability (Figure 2E). These results show that strong evidence is needed for our test to reject the binomial null hypothesis, as expected, and that technical confounders are not major contributors to detection of RAE in our approach.

To further test the fidelity of our method, we performed a simulation study of the GTEx allelic expression data for all 832 people to test RAE FDRs. The algorithm involved simulating biallelic allelic expression for each gene in each GTEx individual according to observed data (see STAR Methods). We found that after multiple testing correction (q value), significant RAE expression is only detected in the observed data and not in the biallelic simulated data, indicating that false classifications of biallelic genes as random allelic are very rare and well controlled with our method (Figure 2F). Overall, these analyses show that significant genes with RAE detected at the individual and population levels with our method are not due to technical factors or false positives, and therefore the RAE is predominantly biological. However, we cannot determine how the RAE relates to cRME versus dRME or other possible non-genetic random allelic effects. Based on these findings, we next set out to profile and study RAE in human tissues at a population level and test associations with phenotypic variation, evolution, disease, and different possible mechanisms.

Identification of high-confidence random allelic genes in human neural and non-neural tissues

High-confidence RAE genes in the human genome are expected to show reproducible effects across different unrelated individuals. To identify these genes, we first used the above approach to uncover significant RAE genes in each of the 832 GTEx subjects, including 279 females and 553 males. For each gene, we then computed the population frequency of significant RAE as a function of sample size, i.e., the number of GTEx subjects with haplotype-phased ASE data for that gene. This analysis yielded a population frequency distribution of RAE for autosomal

CellPress
OPEN ACCESS

A

Technical Factor	Analysis Level: Individual R^2	Analysis Level: GTEx Population R^2
Gene Expression Level (median)	$.007 \pm 1.2 \times 10^{-4}$	0.02
Gene Expression Level (variance)	$9.2 \times 10^{-4} \pm 4.2 \times 10^{-5}$	0.11
Gene Mappability (median)	$4.7 \times 10^{-4} \pm 3.0 \times 10^{-5}$	3.8×10^{-3}
Gene Length	$2.9 \times 10^{-3} \pm 1.3 \times 10^{-4}$	8.5×10^{-4}
Gene SNP Number (per kb)	$.02 \pm 2.1 \times 10^{-4}$	0.08
eQTL Number	$.04 \pm 1.4 \times 10^{-3}$	8.7×10^{-4}
Tissue Number	$0.05 \pm 1.3 \times 10^{-3}$	0.08
Total Mapped Reads (mean)	$5.2 \times 10^{-4} \pm 3.9 \times 10^{-5}$	—
Total Mapped Reads (variance)	$3.9 \times 10^{-4} \pm 2.8 \times 10^{-5}$	—
Tissue RIN (mean)	$5.5 \times 10^{-4} \pm 3.1 \times 10^{-5}$	—
Tissue RIN (variance)	$5.2 \times 10^{-4} \pm 3.3 \times 10^{-5}$	—

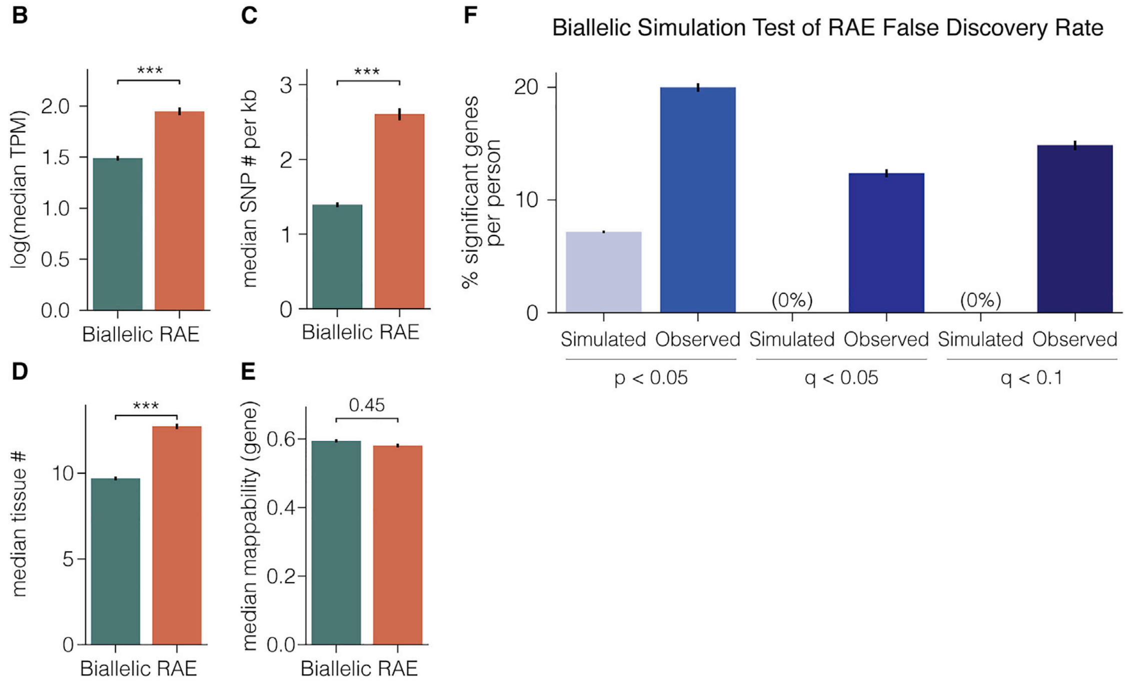

Figure 2. Potential biological and technical factors do not explain the variance in the statistical approach

(A) Table of potential biological and technical factors and the variance explained via logistic regression (R^2) toward the allelic expression test statistic at the individual level (q value) and population level (Z score). Individual R^2 values are represented as the mean ± standard error of the mean (SEM).

(B) Bar plot of median gene expression across tissues (log(median TPM)) for biallelic ($Z < 0$) and RAE ($Z > 0.74$) genes (one-way ANOVA, $p = 1.0 \times 10^{-39}$).

(C) Bar plot of median SNP count per gene (normalized by gene length) for biallelic and RAE genes in the GTEx population (one-way ANOVA, $p = 4.4 \times 10^{-162}$).

(D) Bar plot of median tissue count per gene for biallelic and RAE genes in the GTEx population (one-way ANOVA, $p = 5.6 \times 10^{-178}$).

(E) Bar plot of median 36 bp mappability score per gene for biallelic and RAE genes (Kruskal-Wallis ANOVA, $p = 0.45$).

(F) Simulated binomial null biallelic expression was performed to measure the RAE false discovery rate. We simulated ‘‘true biallelic’’ expression for every gene in each GTEx subject ($n = 832$) by drawing allele counts from the binomial probability distribution generated from the gene’s expression characteristics (i.e., number of tissues, total RNA-seq read counts, and mean allele ratio for all tissues). We then performed the binomial versus beta-binomial hypothesis test for the simulated allelic expression counts and observed allelic expression counts. The bar plot shows the proportion of significant RAE genes (p value or q value) per GTEx individual for the biallelic simulated and observed allele data.

Error bars in (B) to (F) denote SEM. *$p < 0.01$, **$p < 0.001$, ***$p < 0.0001$.

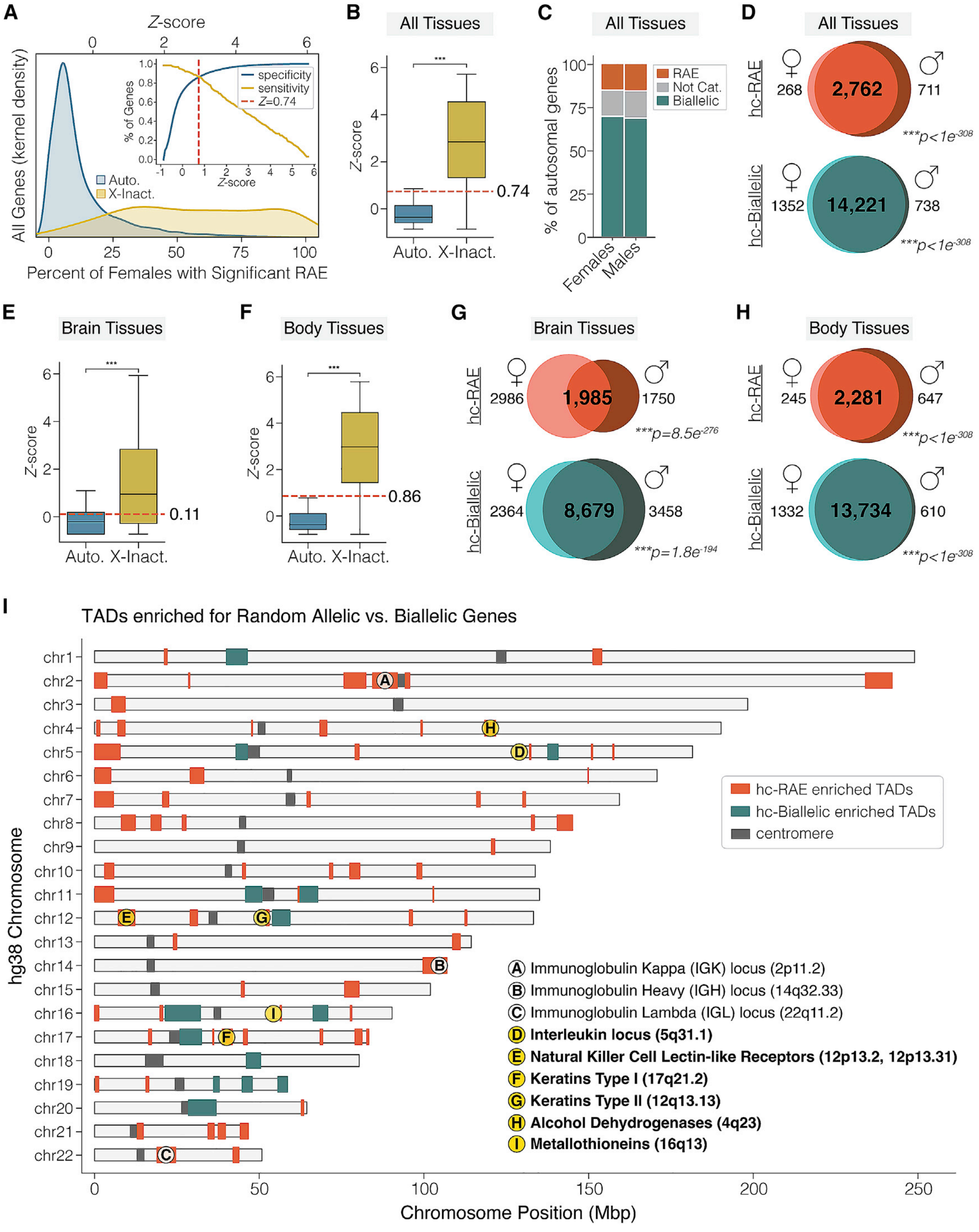

Figure 3. Identifying high-confidence autosomal biallelic and random allelic genes in GTEx and their locations in TADs

(A) The proportion of GTEx females with putative RAE (FDR 10%) for autosomal and XCI genes, plotted as kernel densities. Results were generated using all tissues for each subject. Top x axis shows corresponding *Z* scores. Inset: the sensitivity and specificity of distinguishing XCI (yellow line) versus autosomal (blue line) genes with putative RAE at various *Z*-score cutoffs. Dashed red line shows the optimal cutoff deduced ($Z = 0.74$).

(B) In GTEx females, *Z* scores of autosomal and XCI genes derived from RNA-seq in all tissues (3–41 tissues) (Kruskal-Wallis ANOVA; $p = 1.0 \times 10^{-148}$). Box plot shows median, interquatile range, and min-max values.

(C) Proportion of genes identified as biallelic ($Z < 0$), RAE ($Z > 0.74$), or not categorized (n.c.) ($0 < Z < 0.74$) for female or male subjects. Females: 13.8% (3,035 genes) RAE; 71.1% (15,624 genes) biallelic; 15.1% (3,310 genes) n.c. Males: 15.5% (3,591 genes) RAE; 68.8% (15,890 genes) biallelic; 15.6% (3,610 genes) n.c.

(D) Venn diagram of RAE (top, $p < 1.0 \times 10^{-308}$) and biallelic (bottom, $p < 1.0 \times 10^{-308}$) genes identified in male and female subjects independently using all tissues (3–41 tissues) (chi-squared test). The intersection reveals hc-RAE and hc-biallelic genes; see Table S2 for gene sets.

(legend continued on next page)

and XCI genes in females, and clear differences are apparent (Figure 3A). We found that many autosomal genes (~66%) showed significant RAE in at least one person, but fewer than 15% of people. The highest population frequency of RAE observed for any gene was 98% for *IGHG2*, a member of the random allelic immunoglobulin family (Figure 3A, blue distribution). Our results show that significant RAE on the autosomes is variable between individuals and most genes are affected in a small number of people, suggesting that RAE occurs dynamically for many genes between tissues or individuals.

In contrast to most autosomal genes, most XCI genes in females show a high population frequency of RAE; several are significant in 100% of tested individuals, and the majority are significant in over 50% (Figure 3A, yellow distribution). This result shows that the stable RAE of XCI genes is reproducible across individuals, as expected. Therefore, to identify high-confidence autosomal RAE genes, we empirically derived the population frequency threshold that best yields XCI-like RAE. To this end, we computed *Z* scores to determine how each gene's RAE population frequency compared with the mean for all autosomal and XCI genes combined. Thus, a gene with a *Z* score above zero has a higher-than-average population frequency of RAE. In contrast, a gene with a *Z* score below zero has a below-average frequency of RAE, indicating relatively high biallelic expression. In our data, the average *Z* score for an autosomal gene is −0.05 versus 2.85 for XCI genes in females, and the two distributions are significantly different (Figure 3B). Next, we used sensitivity and specificity curves for autosomal genes (presumed predominately true negatives) versus XCI genes (true positives) in females to determine the optimal *Z*-score threshold that yields reproducible RAE (Figure 3A, inset). The point at which the two curves converge indicates the empirical threshold to define autosomal genes with RAE properties similar to XCI genes ($Z \geq 0.74$) (Figure 3A, inset). By applying this threshold to the data for females (n = 279) and males (n = 553), we found that 13.8% (3,035 genes) and 15.5% (3,591 genes) of autosomal genes, respectively, exhibit RAE analogous to XCI genes (Figure 3C, orange bars). In addition, we defined genes distinguished by relatively robust biallelic expression in the population ($Z \leq 0$) (Figure 3C, green bar) and left a subset of genes undefined ($0 < Z < 0.74$) (Figure 3C, gray bar). In summary, we found autosomal genes with reproducible RAE properties resembling female XCI and strongly co-expressed biallelic genes in human tissues.

To further enrich for genes with high-confidence random allelic expression (hc-RAE) versus those with high-confidence biallelic expression (hc-Biallelic), we identified the genes with these allelic properties that are independently replicated in both the male and female datasets. We found significant reproducibility between the two datasets, revealing that 74% of autosomal RAE genes and 87% of biallelic genes are consistent between sexes (Figure 3D), constituting a catalog of 2,762 hc-RAE autosomal genes (Figure 3D [top] and Table S2) and 14,221 hc-Biallelic genes (Figure 3D [bottom] and Table S2).

We next applied the above approach to neural and non-neural tissues separately to profile hc-RAE genes in the human brain versus the body. As described above, we identified significant RAE genes in the brain and body for GTEx individuals with ASE data from multiple different brain regions (3–13 regions; females, n = 75; males, n = 187) and those with data from multiple non-neural body tissues (3–30 tissues; females, n = 278; males, n = 548). The *Z*-score sensitivity and specificity curves for the female data revealed the threshold for significant RAE genes in the brain (Figure S2, $Z > 0.11$) versus body tissues (Figure S2, $Z > 0.86$). Again, we found significant reproducibility between males and females, revealing 1,985 and 2,281 hc-RAE genes and 8,679 and 13,734 hc-Biallelic genes in the brain and body, respectively (Figures 3G and 3H; Table S2). Interestingly, we observe that sex differences in RAE are more pronounced in the brain than in body tissues (chi-squared test, $p < 1.0 \times 10^{-308}$). Overall, we defined a catalog of hc-RAE and hc-Biallelic genes in human tissues from our population study of RAE, including the brain versus body tissues separately (Table S2), and found that sex differences for RAE are more pronounced in the human brain.

RAE is enriched in topologically associated domains proximal to telomeres

Some well-understood autosomal RAE genes are clustered within the genome, including random allelic immunoglobulins and T cell receptors,[32] olfactory receptors,[33] and protocadherins.[34] Therefore, we asked whether the hc-RAE and hc-Biallelic genes we uncovered above are clustered in the genome and distinctive genomic locations. To answer this, we tested whether topologically associated domains (TADs) in the human genome are significantly enriched for hc-RAE versus hc-Biallelic genes. Using TAD boundaries previously uncovered from 49 different tissue and cell-line datasets (19,633 total TADs),[35] we found 72 TADs significantly enriched for hc-RAE genes and 14 enriched for hc-Biallelic genes (Figure 3I and Table S3) (see STAR Methods). TADs significantly enriched for hc-RAE genes are located on all 22 human autosomes and are frequently located proximal to telomeres (Figure 3I, orange), while hc-Biallelic enriched TADs are proximal to centromeres (Figure 3I, green). The relative telomeric versus centromeric genomic positioning of hc-RAE and hc-Biallelic enriched TADs are significantly different (Kruskal-Wallis $p = 2.97 \times 10^{-19}$) (Figures S2E and S2F). These results show that autosomal RAE and biallelic gene expression are associated with specific TAD locations in the human genome.

Our hc-RAE enriched TADs revealed both known and previously unreported cases (Table S3). For example, we found

(E and F) *Z* scores of autosomal and XCI genes in GTEx females for (E) brain tissues only (3–13 tissues) (Kruskal-Wallis ANOVA; $p = 5.3 \times 10^{-43}$) and (F) body tissues only (3–28 tissues) (Kruskal-Wallis ANOVA, $p = 1.6 \times 10^{-146}$).

(G and H) Significant reproducibility of RAE (top) and biallelic (bottom) genes identified in male and female subjects independently for (G) brain tissues (RAE, $p = 1.8 \times 10^{-194}$; biallelic, $p = 8.5 \times 10^{-276}$), and (H) body tissues (RAE, $p < 1.0 \times 10^{-308}$; biallelic, $p < 1.0 \times 10^{-308}$) (chi-squared test). The intersections reveal hc-RAE and hc-biallelic genes; see Table S2 for gene sets.

(I) TADs significantly enriched for hc-RAE genes (72 regions, orange) and hc-Biallelic genes (14 regions, teal). hc-RAE enriched TADs containing clustered gene families of interest are annotated A–I.

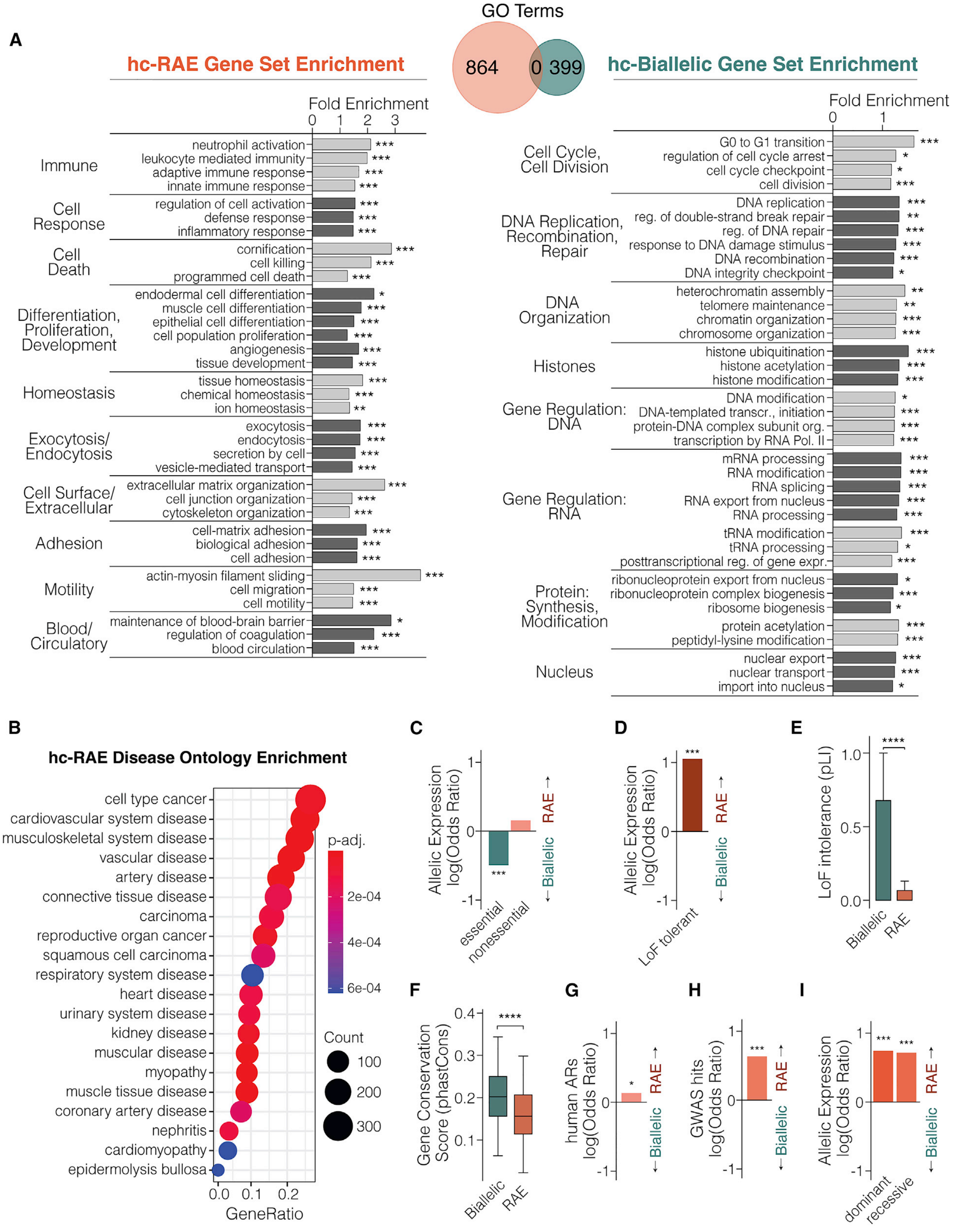

(legend on next page)

CellPress
OPEN ACCESS

enriched TADs harboring the three known random allelic immunoglobulin gene clusters, a positive control for our approach[32,36,37] (Figure 3I). We also identified an interleukin gene-rich TAD and natural killer cell lectin-like receptor gene-rich TAD, which have previously been described as showing variable or stochastic RAE from fluorescent in situ and RT-PCR studies in mouse and human clonal cell lines, but to our knowledge have not been studied *in vivo*[38–43] (Figure 3I). In addition, we identified hc-RAE enriched TADs associated with specific imprinted gene clusters, even though we filtered previously identified human imprinted genes prior to our analysis.[44] Specifically, RAE genes were enriched near the *MEST/PEG1* (7q32.2), *IGF2-H19* (11p15.5), and *DLK1-MEG3* (14q32.2) imprinted gene clusters, revealing epigenetic effects on allele-specific gene expression in these regions that extend beyond canonical imprinting. Unexpectedly, we found multiple hc-RAE enriched TADs that involve gene clusters not previously associated with RAE of interest, including keratin (KRT) genes (17q21.2 and 12q13.3, Figure 3I). Keratins are molecularly diverse intermediate filament proteins that control the integrity and mechanical stability of single epithelial cells, hair follicles, and epithelial tissues, and play important regulatory roles in stress responses, wound healing, and apoptosis.[45,46] We found that keratin type I and type II genes are significantly enriched for hc-RAE in our dataset compared with hc-Biallelic (type I: odds ratio [OR] = 20.59, $p = 4.8 \times 10^{-7}$; type II: OR = 61.79, $p = 1.7 \times 10^{-12}$; chi-squared test). Additionally, we found that the clustered metallothionein (MT) genes (16q13, Figure 3I) are enriched for RAE (OR = 9.01, $p = 1.18 \times 10^{-4}$, chi-squared test). MT genes regulate cellular stress responses, metal ion homeostasis, inflammation, apoptotic responses, and aging.[47] Among other cases (Table S3), we also uncovered hc-RAE for four of the seven clustered alcohol dehydrogenase (ADH) genes in a TAD on chromosome 4q23 (OR = 10.3, $p = 5.22 \times 10^{-3}$, chi-squared test) (Figure 3I). ADH enzymes catalyze the conversion of alcohols to aldehydes and ketones involving the coenzyme nicotinamide adenine dinucleotide (NAD^+) to NADH and the biosynthesis of various metabolites.[48] Allelic diversity among ADH genes in human populations is relatively recent with the advent of rice cultivation and fermentation, and contributes to variation in alcohol metabolism and addiction predisposition between men and women, between young and old, and among geographical locations.[49–51] RAE could be an additional important factor contributing to this variation, as well as evolutionary selection. Our findings show several gene clusters and gene families of interest that exhibit RAE for future study (Table S3).

RAE impacts distinct biological mechanisms compared with biallelic expression and shows links to major age-related diseases, including cancer and neurodegeneration

RAE could be a regulatory feature of genes with specific biological functions and roles in disease processes different from genes with biallelic expression. We tested this by first focusing on the 2,762 hc-RAE and 14,221 hc-Biallelic genes uncovered from all tissues (Figure 3D). We performed Gene Ontology (GO) over-representation analysis for these two gene sets using ClusterProfiler[52] and found 864 and 399 significant GO terms for hc-RAE genes and hc-Biallelic genes, respectively (see STAR Methods) (Figure 4A and Table S4). Strikingly, no significant GO terms are shared between the two gene sets, showing that RAE and biallelic genes are enriched in different pathways (Figure 4A). The top significant molecular pathways for RAE involve adaptive biological response mechanisms, including adaptive and innate immune responses, cellular responses to inflammation, homeostatic processes, changes to cell proliferation, differentiation, development, and apoptosis, and cellular motility and adhesion (Figure 4A). In contrast, the top molecular pathways for biallelic gene expression involve biological processes for essential cellular operations related to the cell cycle, DNA repair, DNA organization, and gene regulation at the level of chromatin, transcription, transcript processing, and protein modifications (Figure 4A). Next, disease ontology (DO) enrichment analysis[52] uncovered 134 DO terms significantly enriched for hc-RAE genes (Figure 4B and Table S5), which revealed significant links to several age-related diseases, including cancer, cardiovascular diseases, musculoskeletal system diseases (e.g., arthritis, osteoporosis, and sarcopenia), kidney disease, and more. We did not identify any significant DO terms for hc-Biallelic genes. Overall, our data show that RAE is enriched among genes involved in homeostatic and adaptive cellular responses and linked to age-related disease processes, while biallelic expression is enriched among genes involved in essential nuclear functions.

Figure 4. Biological functions of hc-RAE and hc-Biallelic genes

(A) Fold enrichments for a subset of significant Gene Ontology (GO) terms for hc-RAE (left) and hc-Biallelic (right) gene sets from all tissues. Venn diagram shows there is no overlap of significant GO terms for either gene set. See Table S4 for full GO results.

(B) Top 15 significant disease ontology (DO) terms for hc-RAE genes identified from all tissues. See Table S5 for full DO results.

(C) Log(odds ratio) of RAE versus biallelic genes deemed essential (683 genes, p = 0.006) or non-essential (913 genes, not significant) in multiple cultured human cell lines using a CRISPR-Cas9 knockout screen.[53]

(D) Genes with at least two different high-confidence LoF variants found in a homozygous state in at least one individual in ExAC (330 genes, Fisher's exact test, $p = 8.86 \times 10^{-12}$).[54]

(E) Gene-level gnomAD v2 pLI scores for biallelic and RAE (Kruskal-Wallis ANOVA, $p = 1.0 \times 10^{-56}$).[55] Error bars show SEM.

(F) PhastCons scores for multiple alignments of 16 primate genomes to the human genome, here plotting the mean phastCons score for each gene (Kruskal-Wallis ANOVA, $p = 3.5 \times 10^{-153}$).[56] Box plot shows median, interquatile range, and min-max values.

(G) log(odds ratio) of the number of RAE and biallelic genes annotated within ±100 kb of human ARs (1,908 ARs, Fisher's exact test, p = 0.01).[57]

(H) Log(odds ratio) of RAE versus biallelic genes nearest to GWAS hits in the NHGRI GWAS catalog (6,636 genes, Fisher's exact test, $p = 1.34 \times 10^{-38}$).[58]

(I) Log(odds ratio) of RAE versus biallelic genes for OMIM disease genes with autosomal dominant (709 genes, Fisher's exact test, $p = 1.16 \times 10^{-9}$) and autosomal recessive inheritance (1,183 genes, Fisher's exact test, $p = 2.2 \times 10^{-12}$).[59,60]

In (B) to (H), adjusted p values, FDR 10%; *p < 0.01, **p < 0.001, ***p < 0.0001.

In a related analysis, we focused on the 1,985 hc-RAE genes identified specifically from brain tissues. GO results revealed that RAE in the brain is most significantly enriched for biological processes involved in neuronal signaling, synaptic functions, and axonal projections (Figure S3A). We uncovered a significant enrichment for processes regulating amyloid-beta formation, revealing that hc-RAE expression impacts Alzheimer's disease risk genes that include *APOE*, *ABCA7*, *CLU*, *NTRK2*, and more (Table S4). Consistent with these results, our DO analysis for hc-RAE genes in the brain found significant enrichments for neurodegenerative diseases, including Alzheimer's disease, tauopathy, and Lewy body dementia, and significant links to mental illnesses and cognitive disorders, including schizophrenia (Figure S3B and Table S5). Biological processes in the brain that are enriched for RAE are distinct from those significantly enriched for hc-Biallelic genes, which involve gene expression regulatory processes including transcription, chromatin, and RNA metabolic processes (Figure S3A and Table S4). Thus, in the human brain, RAE affects neuronal signaling processes compared with fundamental gene regulatory processes for biallelic genes. More generally, hc-RAE genes in all tissues and the brain alone show links to age-related diseases, including cancer, cardiovascular disease, and neurodegenerative disease.

Biallelic expression impacts essential genes, whereas RAE is associated with increased mutation tolerance, evolvability, and links to human phenotypic variation

Diploidy is proposed to have evolved to buffer the effects of partially recessive heterozygous mutations or variants.[61] Therefore, compared with RAE, biallelic gene expression might be enriched for essential genes with increased risks for lethality should a mutation arise. We tested this by first analyzing allelic expression for genes found essential in genome-wide CRISPR-based knockout screens of human cell lines.[53] Consistent with our hypothesis, hc-Biallelic genes are significantly enriched among these essential genes while hc-RAE genes are not (Figure 4C). In contrast, hc-RAE genes are significantly enriched for genes tolerant to homozygous loss-of-function (LoF) variants in humans[54] (Figure 4D). Next, we compared the "probability of loss of function intolerance" (pLI) scores for hc-Biallelic versus hc-RAE gene sets.[55] A lower pLI score indicates increased tolerance to LoF variants, and we found that hc-RAE genes have significantly lower pLI scores than hc-Biallelic genes, revealing that they are more tolerant to LoF variation (Figure 4E). Our results support our hypothesis and show that biallelic expression is enriched among essential and more highly constrained genes, while RAE impacts genes with increased tolerance to mutation and LoF variation.

Gene evolvability is a function of mutation tolerance. We hypothesized that hc-Biallelic genes would also show increased sequence conservation in primates due to links to essential genes and increased genetic constraint compared with hc-RAE genes. To test this, we obtained phastCons genome conservation scores for the alignments of 16 primate genomes to the human genome.[56] The phastCons scores range from 0 to 1 and represent the probability that a nucleotide sequence is under negative selection. We averaged the phastCons scores across the nucleotide sequences for each protein-coding gene in our dataset to obtain gene-level "conservation scores." Consistent with our hypothesis, we found that hc-Biallelic genes have significantly higher conservation scores than hc-RAE genes (Figure 4F). Moreover, human accelerated regions (hARs) indicate genetic elements that are rapidly evolving in humans, and we found that hc-RAE genes are significantly enriched near hARs compared with hc-Biallelic genes (Figure 4G).[57]

Since hc-RAE is enriched for genes that are non-essential, resistant to LoF mutations, rapidly evolving, and linked to later-onset diseases, we hypothesized that hc-RAE genes might play disproportionate roles affecting human phenotypic variation compared with hc-Biallelic genes. In support, we found that published genome-wide association study (GWAS) hits for human phenotypes and disease risks are disproportionately enriched for hc-RAE genes[58] (Figure 4H). In an alternative approach, we tested Online Mendelian Inheritance in Man (OMIM) genes showing either autosomal dominant or recessive inheritance effects on human phenotypes and similarly found that hc-RAE genes are disproportionately enriched among both gene categories[59,60] (Figure 4I). In conclusion, biallelic expression is associated with essential genes, mutation intolerance, and evolutionary conservation, while RAE is associated with increased mutation tolerance, accelerated evolution in the human genome, and genetic loci driving non-lethal human phenotypic variation (Figures 4C–4I).

Subtypes of RAE are revealed from associations with different candidate mechanisms

Diverse forms of RAE have been described in cell lines and isolated primary cells, including clonal versus non-clonal and temporally stable versus dynamic forms.[8] So far, the mechanisms involved are not fully understood. Our approach is expected to capture mechanistically diverse forms of RAE in human tissues. Here, we investigate candidate mechanisms and determine subtypes of RAE genes. We first tested the hypothesis that some forms of RAE involve allele-specific silencing due to transcriptional interference between overlapping genes, which contributes to some forms of genomic imprinting[62] and the random allelic expression of *Xist* and its overlapping antisense gene, *Tsix*, on the X chromosome in females.[63–65] Interestingly, gene overlap is frequent in human and mouse autosomes[66] and possibly rapidly evolving between different species lineages.[67] In the human genome, gene overlap events are more frequent than expected by chance, and sense-antisense gene pairs occur for ~25% of transcripts.[68] Opposite DNA strand overlaps are more likely to interfere with one allele's transcription than same-strand overlaps (Figure 5A). Therefore, we tested whether RAE genes are significantly enriched for opposite-strand gene overlaps compared with biallelic genes. As predicted, the hc-RAE genes found for all tissues and the brain or body only are significantly enriched for opposite-strand gene overlapping (Figures 5B, S4A, and S4B; Table S6). Furthermore, RAE genes are relatively depleted for genes with no overlap, while biallelic genes are more likely to have no overlaps with other genes.

We further tested whether sense-antisense gene overlapping is a conserved feature of RAE and focused on the random allelic genes we previously found in the adult mouse hypothalamus, midbrain, liver, and skeletal muscle.[28] In agreement with our

CellPress
OPEN ACCESS

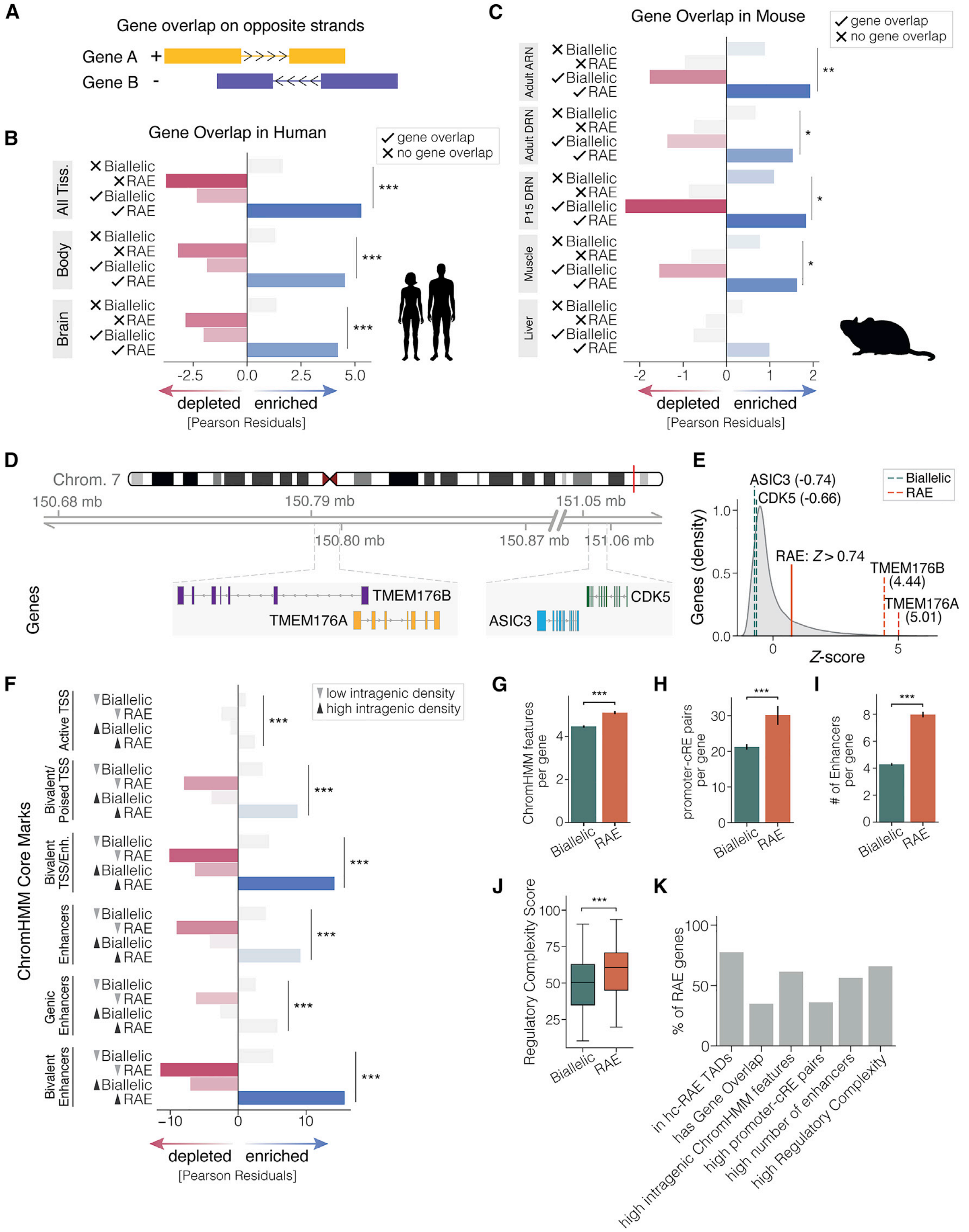

Figure 5. Epigenetic mechanisms underlying RAE in human and mouse

(A) Schematic of how sense-antisense (+/−) gene overlap can lead to transcriptional interference of one allele.

(B) Enrichment or depletion of opposite-strand (+/−) gene overlap in hc-RAE and hc-Biallelic gene sets for all, body, or brain tissues (Pearson chi-square test; all tissues, $p = 1.41 \times 10^{-12}$; body, $p = 2.05 \times 10^{-9}$; brain, $p = 1.77 \times 10^{-8}$).

(C) Enrichment or depletion (Pearson residuals) of opposite-strand gene overlap in RAE versus biallelic genes in P15 and adult mouse brain and body[28] (Pearson chi-squared test; adult ARN, $p = 3.4 \times 10^{-3}$; adult DRN, $p = 0.02$; P15 DRN, $p = 0.01$; adult muscle, $p = 1.0 \times 10^{-3}$; adult liver, $p = 0.16$).

(D) Example of opposite-strand overlapping RAE genes *TMEM176A* and *TMEM176B*, and non-overlapping biallelic genes *CDK5* and *ASIC3*.

(E) Kernel density plot of *Z* scores for all genes (all tissues, male + female subjects) with *Z* scores annotated for genes in (D).

(legend continued on next page)

human data, we found that opposite-strand gene overlap is significantly enriched among RAE genes in mice and was observed for all postnatal and adult brain regions tested and for adult skeletal muscle (Figure 5C). The expected trend was observed in adult mouse liver but did not reach significance ($p = 0.16$). Thus, sense-antisense gene overlapping is a conserved feature of RAE between humans and mice. In one example of this phenomenon in humans, we found that the sense-antisense strand overlapping genes, *TMEM176B* and *TMEM176A*, show RAE, while the neighboring *ASIC3* and *CDK6* genes that do not overlap show biallelic expression (Figures 5D and 5E). Finally, regarding same-strand gene overlaps, there was no significant difference between hc-RAE and hc-Biallelic genes in non-neural tissues in human (Figure S4B) and mouse (Figure S4C), and a modest enrichment was observed for hc-RAE genes in the brain (Figures S4B and S4C; Table S6). Overall, we show that sense-antisense gene overlap is a distinguishing and conserved feature of RAE, impacting approximately 35% of hc-RAE genes in the human genome (Table S6).

The second hypothesis we tested is that genes with RAE are distinguished by an increased density of "intragenic" regulatory features, which could contribute to stochastic variation in allelic expression. Intragenic enhancers have been shown to interfere with host gene transcription.[69] To test differences in the intragenic regulatory features of hc-RAE versus hc-Biallelic genes, we used the published ChromHMM datasets of putative functional *cis* elements, including active transcription start sites (TSS), genic enhancers, enhancers, bivalent/poised TSS, flanking bivalent TSS/enhancers, and bivalent enhancers.[70] We found that RAE genes have a significantly higher intragenic density of each of the different ChromHMM *cis*-regulatory elements (*c*REs) tested than biallelic genes (Figure 5F and Table S7). By analyzing intragenic density, we controlled for potential differences in gene size. RAE genes also have a significantly higher absolute number and diversity of different ChromHMM regulatory elements within the gene body than biallelic genes (Figure 5G). Overall, 61.4% of hc-RAE genes have a higher diversity of intragenic ChromHMM regulatory elements than the median for hc-Biallelic genes. Thus, as predicted, increased density and number of intragenic regulatory elements are distinguishing features of RAE.

Some forms of RAE in isolated cells have been shown to be dynamic, such that cells switch from the expression of one allele to the other.[8] This suggests that each allele may be able to respond to different signals and play independent roles. Decoupling the regulation of each allele could be advantageous for increasing capabilities to respond to and encode different cellular signals, as compared with biallelic genes that express both alleles similarly. Therefore, we tested whether hc-RAE genes have increased *cis*-regulatory inputs compared with hc-Biallelic genes and investigated the numbers of distal *c*REs that contact hc-RAE gene promoters compared with hc-Biallelic genes using published *c*RE-promoter contact maps for 27 human cell/tissue types.[71] We found that the promoters of hc-RAE genes make significantly more promoter-*c*RE contacts than hc-Biallelic genes (Figure 5H). We tested whether this effect was a possible side effect of RAE genes being active in more tissues by also calculating the number of promoter-*c*RE contacts per tissue to control for sampling bias. After controlling for this, hc-RAE genes still make significantly more promoter-*c*RE contacts (Figure S4E). Finally, using the GeneHancer database, a genome-wide integration of enhancers and their target genes,[72] we found that hc-RAE genes have a significantly higher number of enhancers compared with hc-Biallelic genes (Figures 5I and S4F). On average, hc-RAE genes are regulated by 8.7 enhancers versus 4.7 enhancers for hc-Biallelic genes, consistent with our prediction.

To summarize the net regulatory inputs on a given gene, we computed a "regulatory complexity score" for each gene that provides a genome-wide ranking of the complexity of regulatory inputs to a gene (see STAR Methods). As expected, we found that hc-RAE genes have significantly higher regulatory complexity scores, indicating more complex gene regulatory landscapes than hc-Biallelic genes (Figure 5J). We identified that 69.1% of hc-RAE genes have a regulatory complexity score greater than the average hc-Biallelic gene, indicating hc-RAE genes that are candidates for decoupling allelic expression and regulation to increase information encoding (Table S8). Overall, our study uncovered putative subtypes of hc-RAE genes based on (1) location in telomeric TADs, (2) sense-antisense gene overlapping, (3) high-density intragenic regulatory elements, and (4) elevated *cis*-regulatory input complexity (Figure 5K).

DISCUSSION

Our study developed and demonstrated a method to profile RAE in human tissues and uncover high-confidence random allelic and biallelic expressing genes. We found that ~10% of autosomal genes in male and female adults show RAE with some properties resembling random X-inactivation in females, identifying 2,762 hc-RAE genes from all major human tissues and 1,985 in the brain. These genes show significant RAE in

(F) Pearson residuals show enrichment or depletion of intragenic densities of ChromHMM core chromatin states for RAE and biallelic genes. Genes were grouped as either high or low density for each state (top or bottom 50th percentile, respectively) before being grouped by allelic subtype (Pearson chi-squared test, ***$p < 0.0001$). See Table S6 for full results.
(G) Number of different ChromHMM core chromatin states per gene for biallelic versus RAE genes (one-way ANOVA, $p = 5.6 \times 10^{-94}$).
(H) Number of promoter-chromatin (PC) regulatory contacts for promoters of biallelic versus RAE genes (one-way ANOVA, $p = 1.2 \times 10^{-6}$).
(I) Number of GeneHancer enhancers for biallelic versus RAE genes (one-way ANOVA, $p = 1.02 \times 10^{-171}$).
(J) Regulatory complexity scores for biallelic versus RAE genes (one-way ANOVA, $p = 2.5 \times 10^{-85}$). Box plot shows median, interquatile range, and min-max values.
(K) Bar plot depicting the proportion of hc-RAE genes defined by mechanistic subtypes. From left to right: first bar, percentage of hc-RAE genes located within hc-RAE TAD regions (see Figure 2I); second bar, percentage of hc-RAE genes with sense-antisense gene overlap (see Figure 4B); bars 3–6, percentage of hc-RAE genes with values greater than the average hc-Biallelic gene for number of promoter-cRE pairs, enhancers, and regulatory complexity scores (corresponding to G–J). See Table S8 for full results.
Error bars in (G) to (I) denote SEM; *$p < 0.01$, **$p < 0.001$, ***$p < 0.0001$.

individuals, occur frequently in the population cohort, and are reproducible between males and females. Control studies show that hc-RAE is not attributable to genetic *cis*-eQTL effects, low expression level, low mappability, poor resolution of ASE, false discoveries, or other confounders. Instead, distinctive biological properties are associated with hc-RAE versus hc-Biallelic expression. Hc-RAE is enriched in TADs located near telomeres and impacts genes significantly linked to adaptive cellular processes, immunity, homeostasis, and late-onset diseases. These genes largely have non-essential functions, increased mutation tolerance, are enriched for accelerated evolution in humans, and disproportionately impact loci that are significant drivers of human phenotypic variation. In contrast, hc-Biallelic expression is enriched in TADs near centromeres and is associated with essential, mutation intolerant, and more conserved genes. Hc-Biallelic genes are involved in fundamental cellular processes such as DNA organization, gene regulation, and the cell cycle, and do not show significant DO enrichments. Finally, our results delineate candidate mechanistic subtypes of human hc-RAE based on associations with sense-antisense gene overlaps, high intragenic regulatory element density, and increased *cis*-regulatory input complexity.

Diverse forms of RAE have been described in cell lines, including cRME, dRME, and different dynamic versus stable modes of allelic expression.[8,13] So far, the mechanisms underlying these different forms and their effects on phenotypic variation are not well defined but are beginning to be elucidated.[25,26,73] RAE can promote cellular heterogeneity and molecular diversity among otherwise genetically identical cell populations. A classic example of this is random allelic exclusion of T cell receptor and immunoglobulin genes via DNA rearrangement to generate lymphocytes with different antigen-recognition capabilities for adaptive immunity. More stable forms of autosomal RAE may interact with genetic factors in ways that are similar to X-inactivation skewing in females, such that a bias to express a mutant allele in more cells could affect phenotypic variation and increase disease risks.[9–11] On the other hand, dynamic RAE that enables each allele to function independently could increase information encoding, cellular diversity, and the diversity of cellular and molecular responses available within a tissue for adapting to stresses and unpredictable environmental cues. Here, we found putative mechanistic subtypes of RAE in adult human tissues that could point to different forms of RAE. Gene overlapping was revealed to be a conserved feature of RAE in humans and mice and is a candidate for stable RAE given that gene overlapping has been linked to some forms of genomic imprinting,[62] a stable form of monoallelic expression. Additionally, some RAE genes were distinguished by increased intragenic regulatory element density and complexity. If increased regulatory complexity is associated with a decrease in the probability of regulating both alleles similarly in a cell, this could contribute to cellular genetic and gene dose diversity via stochastic and dynamic allelic expression. Intriguingly, we found that hc-RAE genes are enriched in pathways mediating adaptive cellular processes, suggesting important roles in cellular responses to unpredictable environmental signals. Indeed, evolution shows how diversity is essential for populations to survive and adapt to stressors.[74]

Different forms and mechanistic subtypes of RAE in human tissues could interact with genetic factors to influence disease risks and progression. Both dynamic and stable forms of RAE could be important mechanisms in cancer.[75] Thus, our finding that hc-RAE in human tissues is significantly enriched in cancer pathways supports expanded studies of hc-RAE genes in oncogenesis, metastasis, and the evolution of drug resistance. Moreover, our methodology will enable direct profiling of RAE in tumor tissue samples to investigate these relationships, and previous work has shown that monoallelic expression is tumor and gene specific and an important predictor of cancer progression.[76] Importantly, RAE could play related, but advantageous roles in aging and degenerative processes by increasing cellular molecular diversity and, in turn, the ability to adapt to stress and maintain homeostasis within tissues. Thus, the significant links we found between human RAE and neurodegenerative disease genes, as well as genes linked to other age-related diseases, indicate a future direction for the field. By defining the dynamic versus stable RAE genes in a person, and the genetic variants associated, we might predict an individual's potential for having high versus low cellular allelic diversity and adaptability and improve capabilities to predict the impacts of deleterious mutations. For example, we found that the *APOE* gene shows hc-RAE heterozygous *APOE4* allele carriers are known to have increased risks for late-onset Alzheimer's disease,[77] but we do not yet know how RAE may influence these risks. Our study shows population-level variability in RAE, such that some individuals show significant RAE for particular genes while others do not. The presence versus absence of RAE for some genes could affect disease risks, be a marker for cellular stress and adaptive biological responses, or, alternatively, reflect a healthy state of stochastic allelic expression and cellular diversity that is impaired or constrained in some individuals.

In our study, hc-RAE genes show increased tolerance to LoF variation, are enriched among non-essential genes, and are associated with GWAS hits for diverse human phenotypes, corroborating some previous findings.[78] These characteristics indicate an elevated potential for genetic diversity among RAE genes and suggest important roles for non-lethal phenotypic variation. The exposure of genetically different alleles via monoallelic expression can also impose selective pressures on functionally advantageous or disadvantageous gene copies, suggesting that they also shape the selection of genetic variants in a population. In support, we found that hc-RAE genes are less genetically conserved, are enriched near rapidly evolving hARs, and are significantly proximal to telomeres, which are among the most rapidly evolving regions of the genome.[79] Gene families enriched for hc-RAE include keratin gene clusters, which underwent evolutionary bursts of expansion to support the adaptation of animal body plans and epithelial cells to different environments,[45,46] as well as alcohol dehydrogenase gene clusters, which rapidly evolve to support adaptations to different diets, toxins, and metabolic pathways.[48,49] In contrast, hc-Biallelic expression is enriched for essential, conserved, and mutation-intolerant genes. This finding is consistent with predictions that diploidy and biallelic expression is important for some loci to protect against the expression of deleterious mutations.[61,80] Overall, the interplay between RAE and genetically diverse, rapidly

evolving regions suggests that RAE serves as an important mechanism in phenotypic variability and human evolution. One observation in our study is that RAE profiles can differ substantially between different individuals. Some of this variation is caused by variability in the power to detect RAE for a gene between individuals, including the presence versus absence of allele-discriminating SNPs, differences in expression level, and the tissues profiled. However, differences in biological factors could play a role, and future studies can now use our approach to profile and study changes to RAE due to physiological, pharmacological, and disease processes.

Limitations of the study

Our approach to profiling RAE with bulk RNA-seq datasets generated from tissue biopsies does not enable cellular-level resolution of RAE or reveal the temporal dynamics of RAE for particular genes in human tissues. This limits our ability to draw conclusions about the nature of the allelic effect in terms of differentiating clonal RME from other more variable or dynamic forms of RAE. Thus, our approach is designed to detect diverse forms of both dynamic and stable RAE.

STAR★METHODS

Detailed methods are provided in the online version of this paper and include the following:

- KEY RESOURCES TABLE
- RESOURCE AVAILABILITY
  - Lead contact
  - Materials availability
  - Data and code availability
- EXPERIMENTAL MODEL AND SUBJECT DETAILS
- METHOD DETAILS
  - TAD enrichment analysis
  - Gene Ontology enrichment analysis
  - pLI analysis
  - phastCons analysis
  - Human accelerated regions
  - Transcriptional interference (gene overlap) analysis
  - Intragenic regulatory complexity with ChromHMM datasets
  - Promoter-chromatin regulatory contact data
  - Enhancer regulation with GeneHancer datasets
  - Regulatory complexity scores
- QUANTIFICATION AND STATISTICAL ANALYSIS
  - Performing the binomial vs. beta-binomial hypothesis test
  - Technical factors and quality control

SUPPLEMENTAL INFORMATION

Supplemental information can be found online at https://doi.org/10.1016/j.celrep.2022.111945.

ACKNOWLEDGMENTS

We thank members of the Gregg and Quinlan labs for their critical reading of the manuscript and input on the study. Dr. Cornelia Stacher-Hörndli generated the graphical abstract. This work was supported by funding from the National Institutes of Health (R01AG064013, R21MH120468, R01MH109577, and R21MH118570 grants to C.G. and R01HG012252 to A.R.Q.) and fellowships from the National Center for Advancing Translational Sciences of the National Institutes of Health (UL1TR002538) and the University of Utah Graduate Research Fellowship to S.N.K.

AUTHOR CONTRIBUTIONS

S.N.K. developed the methodology and performed the studies; E.F. provided input on the methodology and performed components of the evolution and conservation analysis; A.T. and M.I.L. advised on the statistical methodology; S.N.K., A.R.Q., and C.G. wrote the manuscript with input from all the authors; A.R.Q. and C.G. supervised the study design, methods, and data interpretation and provided funding.

DECLARATION OF INTERESTS

The University of Utah has filed a patent related to this work. C.G. is a co-founder of and has equity in Storyline Health Inc., which uses artificial intelligence to build scalable research and clinical tools for precision medicine.

INCLUSION AND DIVERSITY

We support inclusive, diverse, and equitable conduct of research.

Received: April 14, 2022
Revised: October 17, 2022
Accepted: December 15, 2022

REFERENCES

1. Xue, Y., Chen, Y., Ayub, Q., Huang, N., Ball, E.V., Mort, M., Phillips, A.D., Shaw, K., Stenson, P.D., Cooper, D.N., et al. (2012). Deleterious- and disease-allele prevalence in healthy individuals: insights from current predictions, mutation databases, and population-scale resequencing. Am. J. Hum. Genet. *91*, 1022–1032. https://doi.org/10.1016/j.ajhg.2012.10.015.
2. Bulik-Sullivan, B., Finucane, H.K., Anttila, V., Gusev, A., Day, F.R., Loh, P.R., ReproGen Consortium; Psychiatric Genomics Consortium; Genetic Consortium for Anorexia Nervosa of the Wellcome Trust Case Control Consortium 3; Duncan, L., Perry, J.R.B., Patterson, N., Robinson, E.B., et al. (2015). An atlas of genetic correlations across human diseases and traits. Nat. Genet. *47*, 1236–1241. https://doi.org/10.1038/ng.3406.
3. Brainstorm Consortium; Anttila, V., Bulik-Sullivan, B., Finucane, H.K., Walters, R.K., Bras, J., Duncan, L., Escott-Price, V., Falcone, G.J., Gormley, P., et al. (2018). Analysis of shared heritability in common disorders of the brain. Science *360*, eaap8757. https://doi.org/10.1126/science.aap8757.
4. Gandal, M.J., Haney, J.R., Parikshak, N.N., Leppa, V., Ramaswami, G., Hartl, C., Schork, A.J., Appadurai, V., Buil, A., Werge, T.M., et al. (2018). Shared molecular neuropathology across major psychiatric disorders parallels polygenic overlap. Science *359*, 693–697. https://doi.org/10.1126/science.aad6469.
5. Solovieff, N., Cotsapas, C., Lee, P.H., Purcell, S.M., and Smoller, J.W. (2013). Pleiotropy in complex traits: challenges and strategies. Nat. Rev. Genet. *14*, 483–495. https://doi.org/10.1038/nrg3461.
6. Gratten, J., and Visscher, P.M. (2016). Genetic pleiotropy in complex traits and diseases: implications for genomic medicine. Genome Med. *8*, 78. https://doi.org/10.1186/s13073-016-0332-x.
7. Cummings, B.B., Karczewski, K.J., Kosmicki, J.A., Seaby, E.G., Watts, N.A., Singer-Berk, M., Mudge, J.M., Karjalainen, J., Satterstrom, F.K., O'Donnell-Luria, A.H., et al. (2020). Transcript expression-aware annotation improves rare variant interpretation. Nature *581*, 452–458. https://doi.org/10.1038/s41586-020-2329-2.

8. Kravitz, S.N., and Gregg, C. (2019). New subtypes of allele-specific epigenetic effects: implications for brain development, function and disease. Curr. Opin. Neurobiol. *59*, 69–78. https://doi.org/10.1016/j.conb.2019.04.012.

9. Huppke, P., Maier, E.M., Warnke, A., Brendel, C., Laccone, F., and Gärtner, J. (2006). Very mild cases of Rett syndrome with skewed X inactivation. J. Med. Genet. *43*, 814–816. https://doi.org/10.1136/jmg.2006.042077.

10. Knudsen, G.P.S., Neilson, T.C.S., Pedersen, J., Kerr, A., Schwartz, M., Hulten, M., Bailey, M.E.S., and Ørstavik, K.H. (2006). Increased skewing of X chromosome inactivation in Rett syndrome patients and their mothers. Eur. J. Hum. Genet. *14*, 1189–1194. https://doi.org/10.1038/sj.ejhg.5201682.

11. Samaco, R.C., and Neul, J.L. (2011). Complexities of Rett syndrome and MeCP2. J. Neurosci. *31*, 7951–7959. https://doi.org/10.1523/jneurosci.0169-11.2011.

12. Gimelbrant, A., Hutchinson, J.N., Thompson, B.R., and Chess, A. (2007). Widespread monoallelic expression on human autosomes. Science *318*, 1136–1140. https://doi.org/10.1126/science.1148910.

13. Gendrel, A.-V., Marion-Poll, L., Katoh, K., and Heard, E. (2016). Random monoallelic expression of genes on autosomes: parallels with X-chromosome inactivation. Semin. Cell Dev. Biol. *56*, 100–110. https://doi.org/10.1016/j.semcdb.2016.04.007.

14. Deng, Q., Ramsköld, D., Reinius, B., and Sandberg, R. (2014). Single-cell RNA-seq reveals dynamic, random monoallelic gene expression in mammalian cells. Science *343*, 193–196. https://doi.org/10.1126/science.1245316.

15. Reinius, B., and Sandberg, R. (2015). Random monoallelic expression of autosomal genes: stochastic transcription and allele-level regulation. Nat. Rev. Genet. *16*, 653–664. https://doi.org/10.1038/nrg3888.

16. Reinius, B., Mold, J.E., Ramsköld, D., Deng, Q., Johnsson, P., Michaëlsson, J., Frisén, J., and Sandberg, R. (2016). Analysis of allelic expression patterns in clonal somatic cells by single-cell RNA-seq. Nat. Genet. *48*, 1430–1435. https://doi.org/10.1038/ng.3678.

17. Reinius, B., and Sandberg, R. (2018). Reply to 'High prevalence of clonal monoallelic expression. Nat. Genet. *50*, 1199–1200. https://doi.org/10.1038/s41588-018-0189-6.

18. Larsson, A.J.M., Ziegenhain, C., Hagemann-Jensen, M., Reinius, B., Jacob, T., Dalessandri, T., Hendriks, G.-J., Kasper, M., and Sandberg, R. (2021). Transcriptional bursts explain autosomal random monoallelic expression and affect allelic imbalance. PLoS Comput. Biol. *17*, e1008772. https://doi.org/10.1371/journal.pcbi.1008772.

19. Vigneau, S., Vinogradova, S., Savova, V., and Gimelbrant, A. (2018). High prevalence of clonal monoallelic expression. Nat. Genet. *50*, 1198–1199. https://doi.org/10.1038/s41588-018-0188-7.

20. Borel, C., Ferreira, P.G., Santoni, F., Delaneau, O., Fort, A., Popadin, K.Y., Garieri, M., Falconnet, E., Ribaux, P., Guipponi, M., et al. (2015). Biased allelic expression in human primary fibroblast single cells. Am. J. Hum. Genet. *96*, 70–80. https://doi.org/10.1016/j.ajhg.2014.12.001.

21. Prashant, N.M., Liu, H., Bousounis, P., Spurr, L., Alomran, N., Ibeawuchi, H., Sein, J., Reece-Stremtan, D., and Horvath, A. (2020). Estimating the allele-specific expression of SNVs from 10× genomics single-cell RNA-sequencing data. Genes *11*, 240. https://doi.org/10.3390/genes11030240.

22. Ng, K.K., Yui, M.A., Mehta, A., Siu, S., Irwin, B., Pease, S., Hirose, S., Elowitz, M.B., Rothenberg, E.V., and Kueh, H.Y. (2018). A stochastic epigenetic switch controls the dynamics of T-cell lineage commitment. Elife *7*, e37851. https://doi.org/10.7554/elife.37851.

23. Sands, B., Yun, S., and Mendenhall, A.R. (2021). Introns control stochastic allele expression bias. Nat. Commun. *12*, 6527. https://doi.org/10.1038/s41467-021-26798-4.

24. Lo, C.-A., and Chen, B.E. (2019). Parental allele-specific protein expression in single cells in vivo. Dev. Biol. *454*, 66–73. https://doi.org/10.1016/j.ydbio.2019.06.004.

25. Marion-Poll, L., Forêt, B., Zielinski, D., Massip, F., Attia, M., Carter, A.C., Syx, L., Chang, H.Y., Gendrel, A.-V., and Heard, E. (2021). Locus specific epigenetic modalities of random allelic expression imbalance. Nat. Commun. *12*, 5330. https://doi.org/10.1038/s41467-021-25630-3.

26. Nag, A., Savova, V., Fung, H.-L., Miron, A., Yuan, G.-C., Zhang, K., and Gimelbrant, A.A. (2013). Chromatin signature of widespread monoallelic expression. Elife *2*, e01256. https://doi.org/10.7554/elife.01256.

27. Nag, A., Vigneau, S., Savova, V., Zwemer, L.M., and Gimelbrant, A.A. (2015). Chromatin signature identifies monoallelic gene expression across mammalian cell types. G3 *5*, 1713–1720. https://doi.org/10.1534/g3.115.018853.

28. Huang, W.-C., Ferris, E., Cheng, T., Hörndli, C.S., Gleason, K., Tamminga, C., Wagner, J.D., Boucher, K.M., Christian, J.L., and Gregg, C. (2017). Diverse non-genetic, allele-specific expression effects shape genetic architecture at the cellular level in the mammalian brain. Neuron *93*, 1094–1109.e7. https://doi.org/10.1016/j.neuron.2017.01.033.

29. GTEx Consortium; Ardlie, K.G., Deluca, D.S., Segre, A.V., Sullivan, T.J., Young, T.R., Gelfand, E.T., Trowbridge, C.A., Maller, J.B., Tukiainen, T., et al. (2015). The Genotype-Tissue Expression (GTEx) pilot analysis: multitissue gene regulation in humans. Science *348*, 648–660. https://doi.org/10.1126/science.1262110.

30. Castel, S.E., GTEx Consortium; Mohammadi, P., Aguet, F., Ardlie, K.G., Lappalainen, T., Gabriel, S., Getz, G.A., Graubert, A., Hadley, K., et al. (2020). A vast resource of allelic expression data spanning human tissues. Genome Biol. *21*, 234. https://doi.org/10.1186/s13059-020-02122-z.

31. GTEx Consortium; Laboratory Data Analysis &Coordinating Center LDACC—Analysis Working Group; Statistical Methods groups—Analysis Working Group; Enhancing GTEx eGTEx groups; NIH Common Fund; Jo, B., Mohammadi, P., Park, Y., and Parsana, P.; Biospecimen Collection Source Site—NDRI (2017). Genetic effects on gene expression across human tissues. Nature *550*, 204–213. https://doi.org/10.1038/nature24277.

32. Pernis, B., Chiappino, G., Kelus, A.S., and Gell, P.G. (1965). Cellular localization of immunoglobulins with different allotypic specificities in rabbit lymphoid tissues. J. Exp. Med. *122*, 853–876. https://doi.org/10.1084/jem.122.5.853.

33. Chess, A., Simon, I., Cedar, H., and Axel, R. (1994). Allelic inactivation regulates olfactory receptor gene expression. Cell *78*, 823–834. https://doi.org/10.1016/s0092-8674(94)90562-2.

34. Esumi, S., Kakazu, N., Taguchi, Y., Hirayama, T., Sasaki, A., Hirabayashi, T., Koide, T., Kitsukawa, T., Hamada, S., and Yagi, T. (2005). Monoallelic yet combinatorial expression of variable exons of the protocadherin-α gene cluster in single neurons. Nat. Genet. *37*, 171–176. https://doi.org/10.1038/ng1500.

35. Wang, Y., Song, F., Zhang, B., Zhang, L., Xu, J., Kuang, D., Li, D., Choudhary, M.N.K., Li, Y., Hu, M., et al. (2018). The 3D Genome Browser: a web-based browser for visualizing 3D genome organization and long-range chromatin interactions. Genome Biol. *19*, 151. https://doi.org/10.1186/s13059-018-1519-9.

36. Hozumi, N., and Tonegawa, S. (1976). Evidence for somatic rearrangement of immunoglobulin genes coding for variable and constant regions. Proc. Natl. Acad. Sci. USA *73*, 3628–3632. https://doi.org/10.1073/pnas.73.10.3628.

37. Watson, C.T., and Breden, F. (2012). The immunoglobulin heavy chain locus: genetic variation, missing data, and implications for human disease. Genes Immun. *13*, 363–373. https://doi.org/10.1038/gene.2012.12.

38. Bix, M., and Locksley, R.M. (1998). Independent and epigenetic regulation of the interleukin-4 alleles in CD4+ T cells. Science *281*, 1352–1354. https://doi.org/10.1126/science.281.5381.1352.

39. Held, W., Roland, J., and Raulet, D.H. (1995). Allelic exclusion of Ly49-family genes encoding class I MHC-specific receptors on NK cells. Nature *376*, 355–358. https://doi.org/10.1038/376355a0.

40. Holländer, G.A., Zuklys, S., Morel, C., Mizoguchi, E., Mobisson, K., Simpson, S., Terhorst, C., Wishart, W., Golan, D.E., Bhan, A.K., et al. (1998). Monoallelic expression of the interleukin-2 locus. Science *279*, 2118–2121. https://doi.org/10.1126/science.279.5359.2118.

41. Vance, R.E., Jamieson, A.M., Cado, D., and Raulet, D.H. (2002). Implications of CD94 deficiency and monoallelic NKG2A expression for natural killer cell development and repertoire formation. Proc. Natl. Acad. Sci. USA *99*, 868–873. https://doi.org/10.1073/pnas.022500599.

42. Rhoades, K.L., Singh, N., Simon, I., Glidden, B., Cedar, H., and Chess, A. (2000). Allele-specific expression patterns of interleukin-2 and Pax-5 revealed by a sensitive single-cell RT-PCR analysis. Curr. Biol. *10*, 789–792. https://doi.org/10.1016/s0960-9822(00)00565-0.

43. Raulet, D.H. (2004). Interplay of natural killer cells and their receptors with the adaptive immune response. Nat. Immunol. *5*, 996–1002. https://doi.org/10.1038/ni1114.

44. Baran, Y., Subramaniam, M., Biton, A., Tukiainen, T., Tsang, E.K., Rivas, M.A., Pirinen, M., Gutierrez-Arcelus, M., Smith, K.S., Kukurba, K.R., et al. (2015). The landscape of genomic imprinting across diverse adult human tissues. Genome Res. *25*, 927–936. https://doi.org/10.1101/gr.192278.115.

45. Moll, R., Divo, M., and Langbein, L. (2008). The human keratins: biology and pathology. Histochem. Cell Biol. *129*, 705–733. https://doi.org/10.1007/s00418-008-0435-6.

46. Ho, M., Thompson, B., Fisk, J.N., Nebert, D.W., Bruford, E.A., Vasiliou, V., and Bunick, C.G. (2022). Update of the keratin gene family: evolution, tissue-specific expression patterns, and relevance to clinical disorders. Hum. Genomics *16*, 1. https://doi.org/10.1186/s40246-021-00374-9.

47. Swindell, W.R. (2011). Metallothionein and the biology of aging. Ageing Res. Rev. *10*, 132–145. https://doi.org/10.1016/j.arr.2010.09.007.

48. Thompson, C.E., Freitas, L.B., and Salzano, F.M. (2018). Molecular evolution and functional divergence of alcohol dehydrogenases in animals, fungi and plants. Genet. Mol. Biol. *41*, 341–354. https://doi.org/10.1590/1678-4685-gmb-2017-0047.

49. Carrigan, M.A., Uryasev, O., Frye, C.B., Eckman, B.L., Myers, C.R., Hurley, T.D., and Benner, S.A. (2015). Hominids adapted to metabolize ethanol long before human-directed fermentation. Proc. Natl. Acad. Sci. USA *112*, 458–463. https://doi.org/10.1073/pnas.1404167111.

50. Peng, G.-S., and Yin, S.-J. (2009). Effect of the allelic variants of aldehyde dehydrogenase ALDH2*2 and alcohol dehydrogenase ADH1B*2 on blood acetaldehyde concentrations. Hum. Genomics *3*, 121–127. https://doi.org/10.1186/1479-7364-3-2-121.

51. Walters, R.K., Polimanti, R., Johnson, E.C., McClintick, J.N., Adams, M.J., Adkins, A.E., Aliev, F., Bacanu, S.-A., Batzler, A., Bertelsen, S., et al. (2018). Transancestral GWAS of alcohol dependence reveals common genetic underpinnings with psychiatric disorders. Nat. Neurosci. *21*, 1656–1669. https://doi.org/10.1038/s41593-018-0275-1.

52. Yu, G., Wang, L.-G., Han, Y., and He, Q.-Y. (2012). clusterProfiler: an R Package for comparing biological themes among gene clusters. OMICS A J. Integr. Biol. *16*, 284–287. https://doi.org/10.1089/omi.2011.0118.

53. Hart, T., Tong, A.H.Y., Chan, K., Van Leeuwen, J., Seetharaman, A., Aregger, M., Chandrashekhar, M., Hustedt, N., Seth, S., Noonan, A., et al. (2017). Evaluation and design of genome-wide CRISPR/SpCas9 knockout screens. G3 *7*, 2719–2727. https://doi.org/10.1534/g3.117.041277.

54. Lek, M., Karczewski, K.J., Minikel, E.V., Samocha, K.E., Banks, E., Fennell, T., O'Donnell-Luria, A.H., Ware, J.S., Hill, A.J., Cummings, B.B., et al. (2016). Analysis of protein-coding genetic variation in 60, 706 humans. Nature *536*, 285–291. https://doi.org/10.1038/nature19057.

55. Karczewski, K.J., Francioli, L.C., Tiao, G., Cummings, B.B., Alföldi, J., Wang, Q., Collins, R.L., Laricchia, K.M., Ganna, A., Birnbaum, D.P., et al. (2020). The mutational constraint spectrum quantified from variation in 141, 456 humans. Nature *581*, 434–443. https://doi.org/10.1038/s41586-020-2308-7.

56. Pollard, K.S., Hubisz, M.J., Rosenbloom, K.R., and Siepel, A. (2010). Detection of nonneutral substitution rates on mammalian phylogenies. Genome Res. *20*, 110–121. https://doi.org/10.1101/gr.097857.109.

57. Ferris, E., Abegglen, L.M., Schiffman, J.D., and Gregg, C. (2018). Accelerated evolution in distinctive species reveals candidate elements for clinically relevant traits, including mutation and cancer resistance. Cell Rep. *22*, 2742–2755. https://doi.org/10.1016/j.celrep.2018.02.008.

58. MacArthur, J., Bowler, E., Cerezo, M., Gil, L., Hall, P., Hastings, E., Junkins, H., McMahon, A., Milano, A., Morales, J., et al. (2017). The new NHGRI-EBI Catalog of published genome-wide association studies (GWAS Catalog). Nucleic Acids Res. *45*, D896–D901. https://doi.org/10.1093/nar/gkw1133.

59. Blekhman, R., Man, O., Herrmann, L., Boyko, A.R., Indap, A., Kosiol, C., Bustamante, C.D., Teshima, K.M., and Przeworski, M. (2008). Natural selection on genes that underlie human disease susceptibility. Curr. Biol. *18*, 883–889. https://doi.org/10.1016/j.cub.2008.04.074.

60. Berg, J.S., Adams, M., Nassar, N., Bizon, C., Lee, K., Schmitt, C.P., Wilhelmsen, K.C., and Evans, J.P. (2013). An informatics approach to analyzing the incidentalome. Genet. Med. *15*, 36–44. https://doi.org/10.1038/gim.2012.112.

61. Otto, S.P., and Goldstein, D.B. (1992). Recombination and the evolution of diploidy. Genetics *131*, 745–751.

62. McCole, R.B., and Oakey, R.J. (2008). Unwitting hosts fall victim to imprinting. Epigenetics *3*, 258–260. https://doi.org/10.4161/epi.3.5.7052.

63. Brown, C.J., Ballabio, A., Rupert, J.L., Lafreniere, R.G., Grompe, M., Tonlorenzi, R., and Willard, H.F. (1991). A gene from the region of the human X inactivation centre is expressed exclusively from the inactive X chromosome. Nature *349*, 38–44. https://doi.org/10.1038/349038a0.

64. Ohhata, T., Hoki, Y., Sasaki, H., and Sado, T. (2008). Crucial role of antisense transcription across the Xist promoter in Tsix-mediated Xist chromatin modification. Development *135*, 227–235. https://doi.org/10.1242/dev.008490.

65. Ogawa, Y., Sun, B.K., and Lee, J.T. (2008). Intersection of the RNA interference and X-inactivation pathways. Science *320*, 1336–1341. https://doi.org/10.1126/science.1157676.

66. Ho, M.-R., Tsai, K.-W., and Lin, W.-C. (2012). A unified framework of overlapping genes: Towards the origination and endogenic regulation. Genomics *100*, 231–239. https://doi.org/10.1016/j.ygeno.2012.06.011.

67. Wood, E.J., Chin-Inmanu, K., Jia, H., and Lipovich, L. (2013). Sense-antisense gene pairs: sequence, transcription, and structure are not conserved between human and mouse. Front. Genet. *4*, 183. https://doi.org/10.3389/fgene.2013.00183.

68. Engström, P.G., Suzuki, H., Ninomiya, N., Akalin, A., Sessa, L., Lavorgna, G., Brozzi, A., Luzi, L., Tan, S.L., Yang, L., et al. (2006). Complex loci in human and mouse genomes. PLoS Genet. *2*, e47. https://doi.org/10.1371/journal.pgen.0020047.

69. Cinghu, S., Yang, P., Kosak, J.P., Conway, A.E., Kumar, D., Oldfield, A.J., Adelman, K., and Jothi, R. (2017). Intragenic enhancers attenuate host gene expression. Mol. Cell *68*, 104–117.e6. https://doi.org/10.1016/j.molcel.2017.09.010.

70. Roadmap Epigenomics Consortium; Kundaje, A., Meuleman, W., Ernst, J., Bilenky, M., Yen, A., Heravi-Moussavi, A., Kheradpour, P., Zhang, Z., Wang, J., Ziller, M.J., et al. (2015). Integrative analysis of 111 reference human epigenomes. Nature *518*, 317–330. https://doi.org/10.1038/nature14248.

71. Jung, I., Schmitt, A., Diao, Y., Lee, A.J., Liu, T., Yang, D., Tan, C., Eom, J., Chan, M., Chee, S., et al. (2019). A compendium of promoter-centered long-range chromatin interactions in the human genome. Nat. Genet. *51*, 1442–1449. https://doi.org/10.1038/s41588-019-0494-8.

72. Fishilevich, S., Nudel, R., Rappaport, N., Hadar, R., Plaschkes, I., Iny Stein, T., Rosen, N., Kohn, A., Twik, M., Safran, M., et al. (2017). GeneHancer: genome-wide integration of enhancers and target genes in GeneCards. Database *2017*, bax028. https://doi.org/10.1093/database/bax028.

73. Xu, J., Carter, A.C., Gendrel, A.-V., Attia, M., Loftus, J., Greenleaf, W.J., Tibshirani, R., Heard, E., and Chang, H.Y. (2017). Landscape of monoallelic DNA accessibility in mouse embryonic stem cells and neural progenitor cells. Nat. Genet. *49*, 377–386. https://doi.org/10.1038/ng.3769.

74. Gatenby, R.A., Artzy-Randrup, Y., Epstein, T., Reed, D.R., and Brown, J.S. (2020). Eradicating metastatic cancer and the eco-evolutionary dynamics of anthropocene extinctions. Cancer Res. *80*, 613–623. https://doi.org/10.1158/0008-5472.can-19-1941.

75. Gregg, C. (2021). Starvation and climate change—how to constrain cancer cell epigenetic diversity and adaptability to enhance treatment efficacy. Front. Ecol. Evol. *9*, 693781. https://doi.org/10.3389/fevo.2021.693781.

76. Walker, E.J., Zhang, C., Castelo-Branco, P., Hawkins, C., Wilson, W., Zhukova, N., Alon, N., Novokmet, A., Baskin, B., Ray, P., et al. (2012). Monoallelic expression determines oncogenic progression and outcome in benign and malignant brain tumors. Cancer Res. *72*, 636–644. https://doi.org/10.1158/0008-5472.can-11-2266.

77. Yamazaki, Y., Zhao, N., Caulfield, T.R., Liu, C.-C., and Bu, G. (2019). Apolipoprotein E and Alzheimer disease: pathobiology and targeting strategies. Nat. Rev. Neurol. *15*, 501–518. https://doi.org/10.1038/s41582-019-0228-7.

78. Savova, V., Chun, S., Sohail, M., McCole, R.B., Witwicki, R., Gai, L., Lenz, T.L., Wu, C.T., Sunyaev, S.R., and Gimelbrant, A.A. (2016). Genes with monoallelic expression contribute disproportionately to genetic diversity in humans. Nat. Genet. *48*, 231–237. https://doi.org/10.1038/ng.3493.

79. Baird, D.M. (2018). Telomeres and genomic evolution. Philos. Trans. R. Soc. Lond. B Biol. Sci. *373*, 20160437. https://doi.org/10.1098/rstb.2016.0437.

80. Perrot, V., Richerd, S., and Valéro, M. (1991). Transition from haploidy to diploidy. Nature *351*, 315–317. https://doi.org/10.1038/351315a0.

81. Karimzadeh, M., Ernst, C., Kundaje, A., and Hoffman, M.M. (2018). Umap and Bismap: quantifying genome and methylome mappability. Nucleic Acids Res. *46*, e120. https://doi.org/10.1093/nar/gky677.

82. Quinlan, A.R., and Hall, I.M. (2010). BEDTools: a flexible suite of utilities for comparing genomic features. Bioinformatics *26*, 841–842. https://doi.org/10.1093/bioinformatics/btq033.

83. Durinck, S., Spellman, P.T., Birney, E., and Huber, W. (2009). Mapping identifiers for the integration of genomic datasets with the R/Bioconductor package biomaRt. Nat. Protoc. *4*, 1184–1191. https://doi.org/10.1038/nprot.2009.97.

84. McLean, C.Y., Bristor, D., Hiller, M., Clarke, S.L., Schaar, B.T., Lowe, C.B., Wenger, A.M., and Bejerano, G. (2010). GREAT improves functional interpretation of cis-regulatory regions. Nat. Biotechnol. *28*, 495–501. https://doi.org/10.1038/nbt.1630.

85. Hubisz, M.J., Pollard, K.S., and Siepel, A. (2011). PHAST and RPHAST: phylogenetic analysis with space/time models. Brief. Bioinform. *12*, 41–51. https://doi.org/10.1093/bib/bbq072.

86. Meyer, D., Zeileis, A., and Hornik, K. (2006). The strucplot framework: visualizing multi-way contingency tables with vcd. J. Stat. Soft. *17*. https://doi.org/10.18637/jss.v017.i03.

87. Yee, T.W. (2015). Vector Generalized Linear and Additive Models, with an Implementation in R. https://doi.org/10.1007/978-1-4939-2818-7.

88. Castel, S.E., Mohammadi, P., Chung, W.K., Shen, Y., and Lappalainen, T. (2016). Rare variant phasing and haplotypic expression from RNA sequencing with phASER. Nat. Commun. *7*, 12817. https://doi.org/10.1038/ncomms12817.

89. van de Geijn, B., McVicker, G., Gilad, Y., and Pritchard, J.K. (2014). WASP: allele-specific software for robust discovery of molecular quantitative trait loci. Preprint at bioRxiv. https://doi.org/10.1101/011221.

90. Lieberman-Aiden, E., van Berkum, N.L., Williams, L., Imakaev, M., Ragoczy, T., Telling, A., Amit, I., Lajoie, B.R., Sabo, P.J., Dorschner, M.O., et al. (2009). Comprehensive mapping of long-range interactions reveals folding principles of the human genome. Science *326*, 289–293. https://doi.org/10.1126/science.1181369.

91. Dixon, J.R., Jung, I., Selvaraj, S., Shen, Y., Antosiewicz-Bourget, J.E., Lee, A.Y., Ye, Z., Kim, A., Rajagopal, N., Xie, W., et al. (2015). Chromatin architecture reorganization during stem cell differentiation. Nature *518*, 331–336. https://doi.org/10.1038/nature14222.

92. Rao, S.S.P., Huntley, M.H., Durand, N.C., Stamenova, E.K., Bochkov, I.D., Robinson, J.T., Sanborn, A.L., Machol, I., Omer, A.D., Lander, E.S., et al. (2014). A 3D map of the human genome at kilobase resolution reveals principles of chromatin looping. Cell *159*, 1665–1680. https://doi.org/10.1016/j.cell.2014.11.021.

93. Leung, D., Jung, I., Rajagopal, N., Schmitt, A., Selvaraj, S., Lee, A.Y., Yen, C.-A., Lin, S., Lin, Y., Qiu, Y., et al. (2015). Integrative analysis of haplotype-resolved epigenomes across human tissues. Nature *518*, 350–354. https://doi.org/10.1038/nature14217.

94. Schmitt, A.D., Hu, M., Jung, I., Xu, Z., Qiu, Y., Tan, C.L., Li, Y., Lin, S., Lin, Y., Barr, C.L., et al. (2016). A compendium of chromatin contact maps reveals spatially active regions in the human genome. Cell Rep. *17*, 2042–2059. https://doi.org/10.1016/j.celrep.2016.10.061.

95. Rubin, A.J., Barajas, B.C., Furlan-Magaril, M., Lopez-Pajares, V., Mumbach, M.R., Howard, I., Kim, D.S., Boxer, L.D., Cairns, J., Spivakov, M., et al. (2017). Lineage-specific dynamic and pre-established enhancer–promoter contacts cooperate in terminal differentiation. Nat. Genet. *49*, 1522–1528. https://doi.org/10.1038/ng.3935.

96. Rao, S.S.P., Huang, S.-C., Glenn St Hilaire, B., Engreitz, J.M., Perez, E.M., Kieffer-Kwon, K.-R., Sanborn, A.L., Johnstone, S.E., Bascom, G.D., Bochkov, I.D., et al. (2017). Cohesin loss eliminates all loop domains. Cell *171*, 305–320.e24. https://doi.org/10.1016/j.cell.2017.09.026.

97. Gel, B., and Serra, E. (2017). karyoploteR: an R/Bioconductor package to plot customizable genomes displaying arbitrary data. Bioinformatics *33*, 3088–3090. https://doi.org/10.1093/bioinformatics/btx346.

98. Benjamini, Y., and Hochberg, Y. (1995). Controlling the false discovery rate: a practical and powerful approach to multiple testing. J. Roy. Stat. Soc. B *57*, 289–300. https://doi.org/10.1111/j.2517-6161.1995.tb02031.x.

99. Cotton, A.M., Price, E.M., Jones, M.J., Balaton, B.P., Kobor, M.S., and Brown, C.J. (2015). Landscape of DNA methylation on the X chromosome reflects CpG density, functional chromatin state and X-chromosome inactivation. Hum. Mol. Genet. *24*, 1528–1539. https://doi.org/10.1093/hmg/ddu564.

100. Carrel, L., and Willard, H.F. (2005). X-inactivation profile reveals extensive variability in X-linked gene expression in females. Nature *434*, 400–404. https://doi.org/10.1038/nature03479.

101. Tukiainen, T., Villani, A.-C., Yen, A., Rivas, M.A., Marshall, J.L., Satija, R., Aguirre, M., Gauthier, L., Fleharty, M., Kirby, A., et al. (2017). Landscape of X chromosome inactivation across human tissues. Nature *550*, 244–248. https://doi.org/10.1038/nature24265.

102. Wainer Katsir, K., and Linial, M. (2019). Human genes escaping X-inactivation revealed by single cell expression data. BMC Genom. *20*, 201. https://doi.org/10.1186/s12864-019-5507-6.

103. Balaton, B.P., Cotton, A.M., and Brown, C.J. (2015). Derivation of consensus inactivation status for X-linked genes from genome-wide studies. Biol. Sex Differ. *6*, 35. https://doi.org/10.1186/s13293-015-0053-7.

STAR★METHODS

KEY RESOURCES TABLE

REAGENT or RESOURCE	SOURCE	IDENTIFIER
**Deposited data**		
GTEx v8 haplotype-phased, WASP-corrected ASE data (phASER_WASP_GTEx_v8_matrix.gw_phased.txt.gz)	The Genotype Tissue Expression Project (GTEx)[30]	https://gtexportal.org/home/datasets
GTEx v8 eGene and significant variant-gene associations (GTEx_Analysis_v8_eQTL.tar)	The Genotype Tissue Expression Project (GTEx)[30]	https://gtexportal.org/home/datasets
GTEx v8 median gene-level TPMs by tissue (GTEx_Analysis_2017-06-05_v8_RNASeQCv1.1.9_gene_median_tpm.gct.gz)	The Genotype Tissue E xpression Project (GTEx)[30]	https://gtexportal.org/home/datasets
GRCh38 Gene Model based on Gencode 26 transcript model (gencode.v26.GRCh38.genes.gtf)	The Genotype Tissue Expression Project (GTEx)[30]	https://gtexportal.org/home/datasets
GTEx v8 Allele Specific Expression (ASE) tables	The Genotype Tissue Expression Project (GTEx)[30]	dbGaP accession phs000424.v8.p2
GTEx v8 Sample Attributes	The Genotype Tissue Expression Project (GTEx)[30]	dbGaP accession phs000424.v8.p2
TAD Data Sets (hg38)	3D Genome Browser[35]	http://3dgenome.fsm.northwestern.edu/downloads/hg38.TADs.zip
ChromHMM Core 15-state model (127 epigenomes)	Roadmap Epigenomics Project	https://egg2.wustl.edu/roadmap/data/byFileType/chromhmmSegmentations/ChmmModels/coreMarks/jointModel/final/
gnomAD v2.1.1 Loss-of-Function results (pLOF)	Genome Aggregation Database (gnomaAD); Karczewski et al.[55]	https://storage.googleapis.com/gcp-public-data–gnomad/release/2.1.1/constraint/gnomad.v2.1.1.lof_metrics.by_gene.txt.bgz
phastCons 17-way conservation scores (primates)	UCSC Genome Browser; Pollard et al.[56]	http://hgdownload.cse.ucsc.edu/goldenPath/hg38/phastCons17way/hg38.phastCons17way.bw
Human-centered 100-way multi-alignment	UCSC Genome Browser	http://hgdownload.cse.ucsc.edu/goldenPath/hg19/multiz100way
Human genome mappability track (hg38, 36bp)	UCSC Genome Browser; Karimzadeh et al.[81]	http://hgdownload.soe.ucsc.edu/gbdb/hg38/hoffmanMappability/k36.Bismap.MultiTrackMappability.bw
Promoter-Chromatin Regulatory Contact Data	Jung et al.[71]	N/A
GeneHancer Regulatory Interactions (double elite) (hg38)	UCSC Genome Browser[72]	https://genome.ucsc.edu/cgi-bin/hgTables
**Software and algorithms**		
Bedtools v2.29.1	Quinlan et al.[82]	https://github.com/arq5x/bedtools2
BiomaRt v2.46.3	Durinck et al.[83]	http://bioconductor.org/packages/release/bioc/html/biomaRt.html
ClusterProfiler v3.18.1	Yu et al.[52]	https://bioconductor.org/packages/release/bioc/html/clusterProfiler.html
GREAT v4.0.4	McLean et al.[84]	http://bejerano.stanford.edu/great/public/html/
Karyopype v0.1.6	Jake VanCampen	https://pypi.org/project/karyopype/
Rphast v1.6	Hubisz et al.[85]	https://github.com/CshlSiepelLab/RPHAST
VCD v1.4-9	Meyer et al.[86]	https://cran.r-project.org/web/packages/vcd/index.html
VGAM v1.1-5	Thomas Yee[87]	https://rdrr.io/cran/VGAM/
Random allelic expression testing from bulk RNA-Seq data	This study	Zenodo ID: 10.5281/zenodo.7433661

RESOURCE AVAILABILITY

Lead contact

Further information and requests for resources and reagents should be directed to and will be fulfilled by the lead contact, Christopher Gregg (chris.gregg@neuro.utah.edu).

Materials availability

This study did not generate new, unique reagents.

Data and code availability

- The GTEx v8 data are available from gtexportal.org/home/datasets/.
- The custom code developed in this study is available from Github: https://github.com/snkravitz/human-rae-manuscript (Zenodo ID: 10.5281/zenodo.7433661).
- Any additional information required to reanalyze the data reported in this work is available from the lead contact upon request.

EXPERIMENTAL MODEL AND SUBJECT DETAILS

Our study uses the recently published haplotype-level ASE data from the Genotype Tissue Expression (GTEx) v8 release (dbGaP accession phs000424.v8.p2).[30] All GTEx samples were collected from post-mortem biopsies (fresh, PAXgene preserved tissue samples). Detailed sample collection information and SOPs can be found here: https://gtexportal.org/home/methods#staticTextSampleCollection. This haplotype-level GTEx dataset comprises 15,253 samples from 54 human tissues from 838 individuals, resulting in 153 million haplotype-level AE measurements (Figure 1A). In short, haplotype-level data for all GTEx RNA-seq libraries per subject was generated using phASER v1.0.1, using read-backed phased genotype information derived from whole-genome sequencing.[88] This results in a Gene x Tissue count matrix for each subject comprising a "HAP_A_COUNT|HAP_B_COUNT" (Figure 1A). We used the dataset aligned with allelic mapping bias correction using WASP[89] and subsets only haplotypes that could be phased genome-wide, such that haplotype assignment is consistent across all genes within an individual (file phASER_WASP_GTEx_v8_matrix.gw_phased.txt.gz). As a result, genome-wide, the A|B haplotype assignment refers to the same ancestral allele.

For our analyses, we further filtered loci with a total read depth <10, genes expressed in <3 tissues per subject, and genes with loci flagged for mapping bias or low mappability (Figure S1A). While this reduces the number of genes in our analysis (range of 20364–25544 genes), it reduces false positive RAE predictions owing to biased RNA-seq read mapping and other technical artifacts.

Further information on the GTEx sample collection and subjects such as age, ancestry, cause of death, RNA sample quality, and other phenotypic or sample information are available through the GTEx portal (https://gtexportal.org/home/tissueSummaryPage).

METHOD DETAILS

TAD enrichment analysis

To identify enrichments of RAE and biallelic genes in TADs, we downloaded TAD datasets in human genome version hg38 from the 3D Genome Browser.[35] These datasets are compiled from 46 different human cell line and tissue samples[90–96] For each dataset, we used *bedtools intersect* to annotate the genes in our dataset to TADs.[82] Next, we tallied RAE and Biallelic genes in each domain and calculated the odds ratio (OR) and p values of both RAE and biallelic gene enrichments per TAD (Chi-Squared Test). We corrected p values for multiple testing (Benjamini-Hochberg method) and identified significant TADs (FDR 10%) with an OR $\geq$ 1.5. Finally, we merged and counted overlapping significant TAD boundaries across all datasets using *bedtools merge*. Significant TADs were plotted using the R package karyoploteR[97] (Figure 3I).

Gene Ontology enrichment analysis

Gene Ontology enrichment analyses were performed using the R package ClusterProfiler *enrichG O*.[52] We subset the gene sets (hc-RAE and hc-Biallelic) for only the genes shared by male and female GTEx subjects. The background for each analysis consisted of all genes in the dataset expressed for the relevant tissue types. We excluded all X chromosome genes, known imprinted genes, and HLA locus genes for enrichment analyses. The parameters used for the *enrichGO* method were as follows:

```
ont = "ALL",
minGSSize = 3,
maxGSSize = 3000,
pvalueCutoff = .05,
qvalueCutoff = 0.1,
OrgDb = org.Hs.eg.db,
pAdjustMethod = "BH",
```

```
readable = TRUE,
pool = TRUE
```

Results

- All Tissues: RAE (864 significant GO terms); Biallelic (399 significant GO terms)
- Brain Tissues: RAE (217 significant GO terms); Biallelic (57 significant GO terms)
- Body Tissues: RAE (834 significant GO terms); Biallelic (473 significant GO terms)

pLI analysis

We downloaded the gnomAD v2.1 dataset containing pLoF metrics by gene derived from 125,748 exomes and 15,708 whole genomes (gnomad.v2.1.1.lof_metrics.by_gene.txt).[55] Next, we annotated the genes in our dataset with the ‘pLI’ column from the gnomAD dataset and removed any genes with no pLI information. Finally, we plotted RAE and biallelic genes’ pLI scores (Figure 4E).

phastCons analysis

We downloaded the phastCons 17-way (primates) conservation scores from the UCSC Genome Browser[56] (http://hgdownload.cse.ucsc.edu/goldenPath/hg38/phastCons17way/hg38.phastCons17way.bw). After converting the BigWig file to BED format, we used bedtools to map phasCons scores to the GTEx genes in our dataset and compute the mean score per gene.[82] We filtered out non-protein-coding genes and plotted the mean phastCons conservation scores for RAE and biallelic genes (Figure 4F).

Human accelerated regions

To define human accelerated regions (hARs), we used functions from the R package rphast v1.6.[81] We downloaded a 100 way, human centered multi-alignment (UCSC: http://hgdownload.cse.ucsc.edu/goldenPath/hg19/multiz100way). We used phastCons to define 50bp autosomal regions conserved in the 21 non-human mammals in the whole genome alignment with the following options: expected.length = 45, target.coverage = 0.3, rho = 0.31. We tested the resulting 153,6548 regions for acceleration in humans relative to the 21 mammal species with phyloP using the following options: mode = ‘ACC’. After correcting for multiple-testing using the q-value method, we defined 1,908 statistically significant hARs with a false discovery rate threshold of 10%. Finally, we tested for enrichment near RAE and Biallelic genes (Figure 4G).

Transcriptional interference (gene overlap) analysis

Human (Figure 5B)

For each dataset (i.e., all tissues, brain, or body), we used *bedtools intersect* to identify genes overlapping other loci by at least 1bp. This analysis was performed to identify opposite strand overlaps (+/−) and same strand overlaps (+/+ and −/−) separately. Gene coordinates and strand information were obtained from a GTF file (gencode.v26.GRCh38.genes.gtf) via the GTEx Portal (https://gtexportal.org/home/datasets), based on the GENCODE 26 transcript model, where isoforms were collapsed to a single transcript per gene.

Overlaps with other loci were collapsed into a Boolean True/False annotation for each gene. We tested whether the number of RAE vs. Biallelic autosomal genes with gene overlap was significantly different using the Pearson chi-squared test of independence. The Pearson residuals from this test revealed the direction and magnitude of any resulting enrichment or depletion compared to expected frequencies.

Mouse (Figure 5C)

To determine whether transcriptional interference via gene overlap is a mechanism conserved in mice, we utilized our previously published dataset identifying biallelic and autosomal random allelic genes in CastEiJ x C57BL6/J F1 hybrid mouse brain and body tissues (Table S1,[28]). In brief, we repeated the above analysis to identify opposite and same strand overlaps and tested for a significant difference between biallelic and RAE autosomal genes. Gene coordinates and strand information were obtained using the biomaRt R package and the “mmusculus_gene_ensembl” dataset for mm10.

X chromosome genes, known imprinted genes, and HLA genes were removed from human and mouse enrichment analyses.

Intragenic regulatory complexity with ChromHMM datasets

To investigate the epigenetic regulatory features associated with RAE and Biallelic expressed genes, we downloaded the ChromHMM core 15-state model BED files (hg38) from Roadmap Epigenomics.[70] ChromHMM is a multivariate hidden Markov model trained on histone modifications (H3K4me3, H3K4me1, H3K36me3, H3K27me3, H3K9me3) to identify 15 chromatin states in 127 epigenomes. Of the 127 annotated epigenomes, we used only the datasets derived from adult primary tissues to best match GTEx tissues. This resulted in 37 adult primary tissue BED files; 8 were derived from primary brain tissues and 29 non-brain tissues.

We used bedtools *unionbedg* to get the union of all chromatin states per bp genome-wide for all tissues, brain, or body only. Next, we used bedtools *intersect* to annotate per-bp coverage of each of the 15 chromatin states to genes.[82] We calculated the density of each chromatin state per gene to control for gene length. We designated “low density” and “high density” genes for each chromatin state. “Low density” genes had a per-bp density less than the genome-wide median, while “high density” genes had densities greater than the median. We counted the number of RAE and biallelic genes in the low and high-density groups and performed a Pearson’s chi-squared test of independence to test whether there was an enrichment or depletion of any chromatin state in RAE and Biallelic genes.

Promoter-chromatin regulatory contact data

To understand how regulatory connections for promoters of RAE and biallelic genes differ and how their behavior may be shaping allelic expression in human tissues, we downloaded gene - *cis*-regulatory element interactions in Table S3 from Jung et al. 2019.[71] In this paper, the authors generated a map of long-range chromatin interactions centered on 18,943 well-annotated promoters for protein-coding genes in 27 human cell and tissue types and inferred the target genes of 70,329 candidate *c*REs.

We downloaded BED files of cell/tissue-specific cRE-promoter pairs connected by pcHi-C interactions. We used the cRE-promoter pairs derived from Hi-C experiments performed on adult primary tissues to best match GTEx tissues (20 tissues). We counted the number of cREs per gene for each tissue for genes in our dataset. The mean number of PC-interactions per tissue is shown in Figure 5H; error bars are the standard error of the mean.

Enhancer regulation with GeneHancer datasets

To evaluate gene-enhancer regulation, we utilized the GeneHancer Regulatory Interactions (double elite) datasets for hg38 autosomes downloaded from the UCSC genome browser[72] (https://genome.ucsc.edu/cgi-bin/hgTables). GeneHancer is a database annotating regulatory elements to their inferred target genes based on distance information and molecular data; the "double elite" dataset comprises a highly filtered subset of elements and interactions.

We annotated enhancers to their gene interactions in our dataset for our analyses. We quantified the number of enhancers annotated to each gene to generate the barplot in Figure 5I. Error bars indicate the standard error of the mean.

Regulatory complexity scores

To generate a Regulatory Complexity Score for each gene, we compiled the following information from analyses previously described:

- number of 6 core chromatin states (intragenic) (ChromHMM)[70]
- per-base pair density of 6 core chromatin states (intragenic) (ChromHMM)[70]
- mean number of promoter-cRE interactions per tissue[71]
- number of enhancers per gene (GeneHancer)[72]

We next generated percentile ranks for each of the above metrics for each gene. Finally, we developed a "Regulatory Complexity Score" by calculating the median percentile for all metrics.

QUANTIFICATION AND STATISTICAL ANALYSIS

Identifying different subtypes of allelic expression involves first quantifying the expression of a gene's two alleles. In brief, this is achieved by counting RNA-seq reads overlapping heterozygous single-nucleotide polymorphisms (SNPs) for a given gene. These measurements capture the potential effects of genetic or epigenetic *cis*-regulatory variation on either allele's expression. Our previous methodology to profile non-genetic allelic effects was optimized for F1 hybrid inbred mice (C57BL6/J x CAST/EiJ), in which we could sum reads across SNPs derived from the maternal or paternal allele.[28] Humans, however, are not genetically identical; human genetic variation is vast and diverse such that summing RNA-seq reads across multiple SNPs can become complex and introduce inaccuracies. Previous studies have utilized trios to phase haplotypes across multiple SNPs or measure ASE from a single SNP per gene. However, the former approach limits study size, and the latter can introduce technical biases such as coverage or mapping bias.

Performing the binomial vs. beta-binomial hypothesis test

For each GTEx subject, we generate two data matrices: "Allele 1" counts and "Total" allele counts (HAP_A_COUNT and HAP_A_COUNT + HAP_B_COUNT, respectively, in the phASER_WASP_GTEx_v8_matrix.gw_phased.txt.gz data file), where the rows are genes, and the columns are tissues. Because this dataset contains genome-wide haplotype phased counts, we expect these probabilities, or allele ratios, correspond to the same ancestral allele for every gene and every tissue for that individual. For each gene (*i*), we use the observed allele counts (*X*) and total counts (*N*) represent a vector of values for the counts from all tissues to calculate the probability (*p*) of sequencing that allele, or haplotype.

For example, for $Gene_i$ expressed in five tissues:
Tissues = [Liver, Stomach, Heart, Lung, Kidney]
"Allele 1" counts (*X*): = [7, 8, 46, 32, 17]
"Total" counts (*N*): = [14, 16, 90, 65, 35]
$p = sum(X)/sum(N) = 0.5$.

The different tissue samples are derived from the same individual and, therefore, the same genetic background. While a gene's total expression may be higher or lower in different tissues, we expect the allele ratios will be relatively consistent for autosomal genes. Consequently, the null expectation (H_0) is that for a given gene (*i*), the distribution of allele ratios (*X*/*N*) across all the tissue samples will follow a binomial distribution centered at *p*:

$$H_0 : X_i \sim Binomial(N_i, p)$$

If a gene exhibits RAE, such as X chromosome inactivated (XCI) genes, the mosaic monoallelic expression of either allele will result in more variable allele ratios across tissue samples (Figure 1B). For example, Allele 1 may be expressed higher than Allele 2 in some tissues but lower in others. Importantly, this variance will be greater than expected from a binomial distribution and is overdispersed. Consequently, the alternative hypothesis (H_1) is that for a given gene (i), the observed allele ratios in the data are fit by a beta-binomial distribution:

$$H_1 : X_i \sim Beta - Binomial(N_i, \alpha, \beta)$$

We calculate the fit for both hypotheses and perform a one-tailed chi-squared test on the log likelihood of H_1 *vs*. H_0. A significant p value indicates we reject the null hypothesis, and the allele ratios for that gene are overdispersed. We control for multiple testing by applying a Benjamini-Hochberg p value correction for each GTEx subject[98] and use a false-discovery rate (FDR) of 10% to identify significantly overdispersed genes.

To assess the behavior of the test at different expression levels and the number of observations (tissues), we performed simulations around the null (biallelic) expectation. In brief, for each gene, we simulated allele counts (N) from a binomial distribution, where the total counts (N), probability (p), and the number of observations were derived from the actual data. These simulations helped determine a conservative 10% FDR to differentiate significant genes in our datasets.

Technical factors and quality control

To thoroughly examine the utility of our statistical method to enrich for non-genetic effects on allelic expression and identify any potential confounders, we performed several rigorous quality control analyses. These included testing for potential relationships between the outcome of the statistical test and several technical and biological factors at both the individual and population levels within the GTEx cohort. Biological factors include genetic and gene expression characteristics such as overall transcript abundance and variance between tissues, gene length, the number of genetic polymorphisms within the gene body, the number of significant eQTLs for each gene, and the number of tissues a gene is expressed in. Technical factors include the effective library size, measured as the number of uniquely mapped reads, gene mappability scores, and RNA quality across tissue samples.

At the individual level, for each GTEx subject (n = 832) we generated goodness of fit R-squared and p values via linear regression to determine the proportion of variation in the Binomial vs. Beta-Binomial test statistic (B-H corrected q-values) explained by each technical factor. We computed the mean R-squared +/− SEM (standard error of the mean) of all regression fits (Figure 2A). For the population-level values, we calculated the linear regression fit for a gene's population level *Z score* versus each technical factor (Figure 2A).

A brief description of how the data for each technical factor was obtained or generated:

Gene expression

For each GTEx subject, we calculated the median gene expression and variance for every gene according to the tissue samples specific to that subject (GTEx_Analysis_2017-06-05_v8_RNASeQCv1.1.9_gene_tpm.gct.gz). Next, we generated goodness of fit R-squared and p values via linear regression to determine the proportion of variation in the Binomial vs. Beta-Binomial test statistic (q-values) explained by gene expression for each subject (n = 832). We computed the mean R-squared +/− SEM (standard error of the mean) of all regression fits (gene expression mean: $R^2 = 0.007 \pm 1.2 \times 10^{-4}$; expression variance: $R^2 = 9.2 \times 10^{-4}$ +/− 4.2×10^{-5}). To understand the relationship between a gene's expression levels and variance for the population-level frequency of random allelic expression, we generated the linear regression for the *Z* score statistic versus the median ($R^2 = .02$; $p = 3.2x10^{-83}$) and variance ($R^2 = 0.11$; $p < 1x10^{-308}$) gene-level TPMs. These data can be accessed through the GTEx online portal open access downloads (GTEx_Analysis_2017-06-05_v8_RNASeQCv1.1.9_gene_median_tpm.gct.gz).

Gene mappability

We calculated the median multi-read mappability for every gene according to the Bismap 36 bp multi-read mappability scores representing the probability that a randomly selected k-mer (36 bp) that overlaps with a given position is uniquely mappable (UCSC:/gbdb/hg38/hoffmanMappability/k36.Bismap.MultiTrackMappability.bw).[81] We then calculated the linear regression R-squared and p values for median mappability versus gene q-value for each GTEx subject and calculated the mean ± SEM across all subjects (n = 832; $R^2 = 4.7 \times 10^{-4}$ +/− 3.0×10^{-5}). For the population-level values, we calculated the linear regression fit for a gene's *Z score* versus median mappability ($R^2 = 3.8 \times 10^{-3}$).

Gene length

We calculated gene length using the human genome version GRCh38 gene-level model based on the GENCODE 26 transcript model, where isoforms were collapsed to a single transcript per gene (GTEx Portal v8 Downloads, gencode.v26.GRCh38.genes.gtf). We then calculated the linear regression R-squared and p values for gene length versus gene q-value for each GTEx subject and calculated the mean ± SEM across all subjects (n = 832; $R^2 = 2.9 \times 10^{-3}$ +/− 1.3×10^{-4}). For the population-level values, we calculated the linear regression fit for a gene's *Z* score versus gene length ($R^2 = 8.5 \times 10^{-4}$; $p = 2.3x10^{-4}$).

Gene SNP number

For each GTEx subject, we counted the number of heterozygous single nucleotide variants (SNPs) present in the transcribed portion of each gene. We then calculated the linear regression R-squared and p values for SNV number (normalized by gene length) versus

gene q-value for each GTEx subject and calculated the mean ± SEM across all subjects (n = 832; $R^2 = 0.02 \pm 2.1 \times 10^{-4}$). For the population-level values, we calculated the linear regression fit for a gene's *Z score* versus median SNP count across all subjects, normalized per kb of gene length ($R^2 = 0.8$; $p < 1x10^{-308}$).

eQTL number

We counted the total number of unique significant variant-gene associations (eQTLs) for each individual GTEx subject, for each gene. Significant variant-gene associations were obtained from GTEx v8 single-tissue cis-eQTL data (GTEx Portal v8 Downloads; GTEx_Analysis_v8_eQTL.tar). eQTL number was then normalized by median gene expression (TPMs) across tissues before performing the linear regression for variance in q-value (mean ± SEM across all subjects: $R^2 = 0.04 \pm 1.4 \times 10^{-3}$). For the population-level values, we calculated the linear regression fit for a gene's *Z score* versus the median total eQTL count per gene across all subjects, normalized by median gene expression TPMs ($R^2 = 8.7 \times 10^{-4}$; $p = 8.4x10^{-6}$).

Tissue number

For each GTEx subject, we counted the number of tissues for which each gene was expressed, according to the allele-specific expression datasets (GTEx Portal v8 Downloads; phASER_WASP_GTEx_v8_matrix.gw_phased.txt.gz). We then calculated the linear regression R-squared and p values for tissue count versus gene q-value for each GTEx subject and calculated the mean ± SEM across all subjects (n = 832; $R^2 = 0.05 \pm 1.3 \times 10^{-3}$). For the population-level values, we calculated the linear regression fit for a gene's *Z score* versus the median tissue count per gene across all GTEx subjects ($R^2 = 0.08$; $p < 1x10^{-308}$).

Total mapped reads

For each GTEx subject, we obtained the total number of mapped reads for each tissue sample and calculated the mean and variance according to which tissues each gene was expressed in. We then calculated the linear regression R-squared and p values for RIN versus gene q-value for each GTEx subject and calculated the mean ± SEM across all subjects (n = 832; mean mapped reads: $R^2 = 5.2 \times 10^{-4}$ +/− 3.9×10^{-5}; variance mapped reads: $R^2 = 3.9 \times 10^{-4}$ +/− 2.8×10^{-5}). Total mapped reads information obtained from the GTEx online portal (GTEx v8 Downloads; phs000424.v8.pht002743.v8.p2.c1.GTEx_Sample_Attributes.GRU.txt.gz).

Tissue RIN

For each GTEx subject, we obtained the RNA integrity number (RIN) value for each tissue sample and calculated the RIN mean and variance according to which tissues each gene was expressed in. We then calculated the linear regression R-squared and p values for RIN versus gene q-value for each GTEx subject and calculated the mean ± SEM across all subjects (n = 832; mean RIN: $R^2 = 5.5 \times 10^{-4}$ +/− 3.1×10^{-5}; variance RIN: $R^2 = 5.2 \times 10^{-4}$ +/− 3.3×10^{-5}). RIN information was obtained from the GTEx online portal (phs000424.v8.pht002743.v8.p2.c1.GTEx_Sample_Attributes.GRU.txt.gz).

Biallelic expression simulation and GTEx RAE false discovery rate analysis

The algorithm for simulating biallelic expression for each gene in each GTEX individual is provided below. This algorithm was used to estimate false discoveries of RAE in the GTEx data. The code is also provided for the following:

1) Compute total expression of the target gene (*i*) in each tissue for a person (*j*) by summing the observed allelic reads
2) Create a vector of the total expression values (N_{ij}) observed in the tissues for $Gene_{ij}$
3) Simulate biallelic expression for each allele of $Gene_{ij}$ for each tissue by randomly drawing from the binomial distribution based on the observed total expression counts (N_{ij}) and a biallelic expression probability ($p = 0.5$)

$$Allele1_{sim} = rbinom(length(N_{ij}), N_{ij}, p)$$

$$Allele2_{sim} = N_{ij} - Allele1_{sim}$$

4) Compute the fit to the beta-binomial distribution and log likelihood (ll).

$$fit_{betabi} = vglm(cbind(Allele1_{sim}, Allele2_{sim}) \sim 1, family = betabinomial)$$

$$ll_{betabi} = sum(log(dbetabinom(Allele1_{sim}, N_{ij}, mu, rho)))$$

5) Compute the log likelihood for the binomial distribution

$$ll_{bin} = sum(log(dbinom(Allele1_{sim}, N_{ij}, p)))$$

6) Compare the fits and compute the p value testing the biallelic binomial null hypothesis

$$pchisq(2^{*}(ll_{betabi} - ll_{bi}), df1 - df0, lower.tail = F)$$

7) Store the p value for $Gene_i$ in person j

$$PGene_{ij}$$

8) Repeat for each gene (∼20,000 genes) for each person
9) Define number of false significant RAE genes after multiple testing correction (q-value <0.05 and <0.1).
10) Repeat for each GTEx individual (832 people) and estimate the mean population false RAE discovery rate and standard deviation.

Cell Reports, Volume *42*

Supplemental information

Random allelic expression in the adult human body

Stephanie N. Kravitz, Elliott Ferris, Michael I. Love, Alun Thomas, Aaron R. Quinlan, and Christopher Gregg

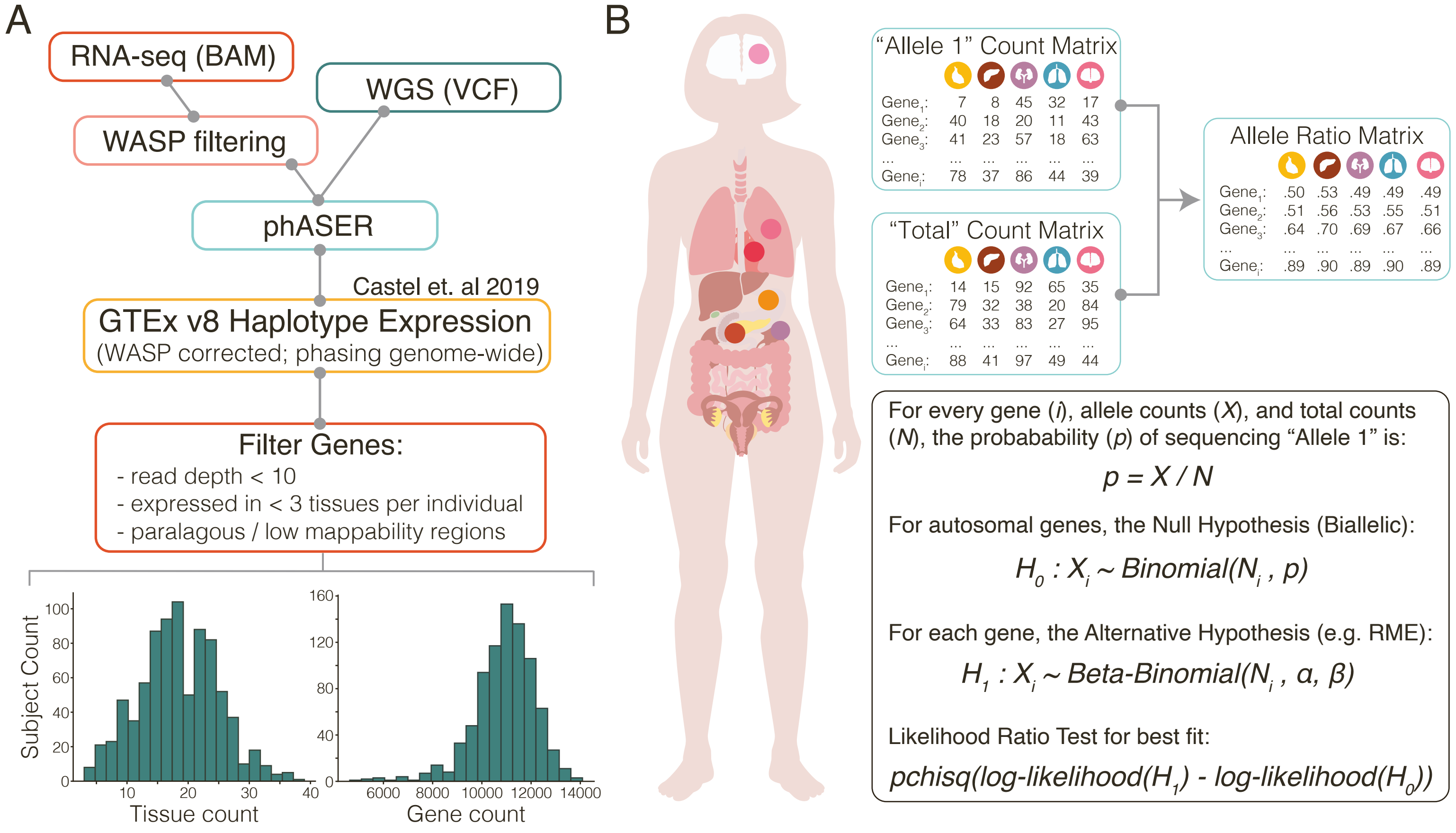

A
RNA-seq (BAM)
WASP filtering
WGS (VCF)
phASER
Castel et. al 2019
GTEx v8 Haplotype Expression
(WASP corrected; phasing genome-wide)
Filter Genes:
- read depth < 10
- expressed in < 3 tissues per individual
- paralagous / low mappability regions
Subject Count
Tissue count
Gene count
B
"Allele 1" Count Matrix
Gene1: 7 8 45 32 17
Gene2: 40 18 20 11 43
Gene3: 41 23 57 18 63
Genei: 78 37 86 44 39
"Total" Count Matrix
Gene1: 14 15 92 65 35
Gene2: 79 32 38 20 84
Gene3: 64 33 83 27 95
Genei: 88 41 97 49 44
Allele Ratio Matrix
Gene1: .50 .53 .49 .49 .49
Gene2: .51 .56 .53 .55 .51
Gene3: .64 .70 .69 .67 .66
Genei: .89 .90 .89 .90 .89
For every gene (i), allele counts (X), and total counts (N), the probability (p) of sequencing "Allele 1" is:
p = X / N
For autosomal genes, the Null Hypothesis (Biallelic):
H0 : Xi ~ Binomial(Ni , p)
For each gene, the Alternative Hypothesis (e.g. RME):
H1 : Xi ~ Beta-Binomial(Ni , α, β)
Likelihood Ratio Test for best fit:
pchisq(log-likelihood(H1) - log-likelihood(H0))

Figure S1. An approach to define genes with biallelic and random monoallelic expression from bulk RNA-Seq data. Related to Figure 1.

(A) Workflow schematic of data processing and filtering of GTEx v8 haplotype-phased ASE datasets (Castel et. al 2019). Bottom plots show the distribution of the number of different tissue datasets available for GTEx subjects in the v8 cohort (left), and the number of genes able to be assessed per subject after filtering (right). (B) Schematic of the multiple tissue RNAseq datasets from a single GTEx subject (left), and the resulting allelic expression data matrices (blue boxes) used to generate the binomial and beta-binomial probability distributions for each gene. A likelihood ratio test is applied for each gene to determine the best fit (black box).

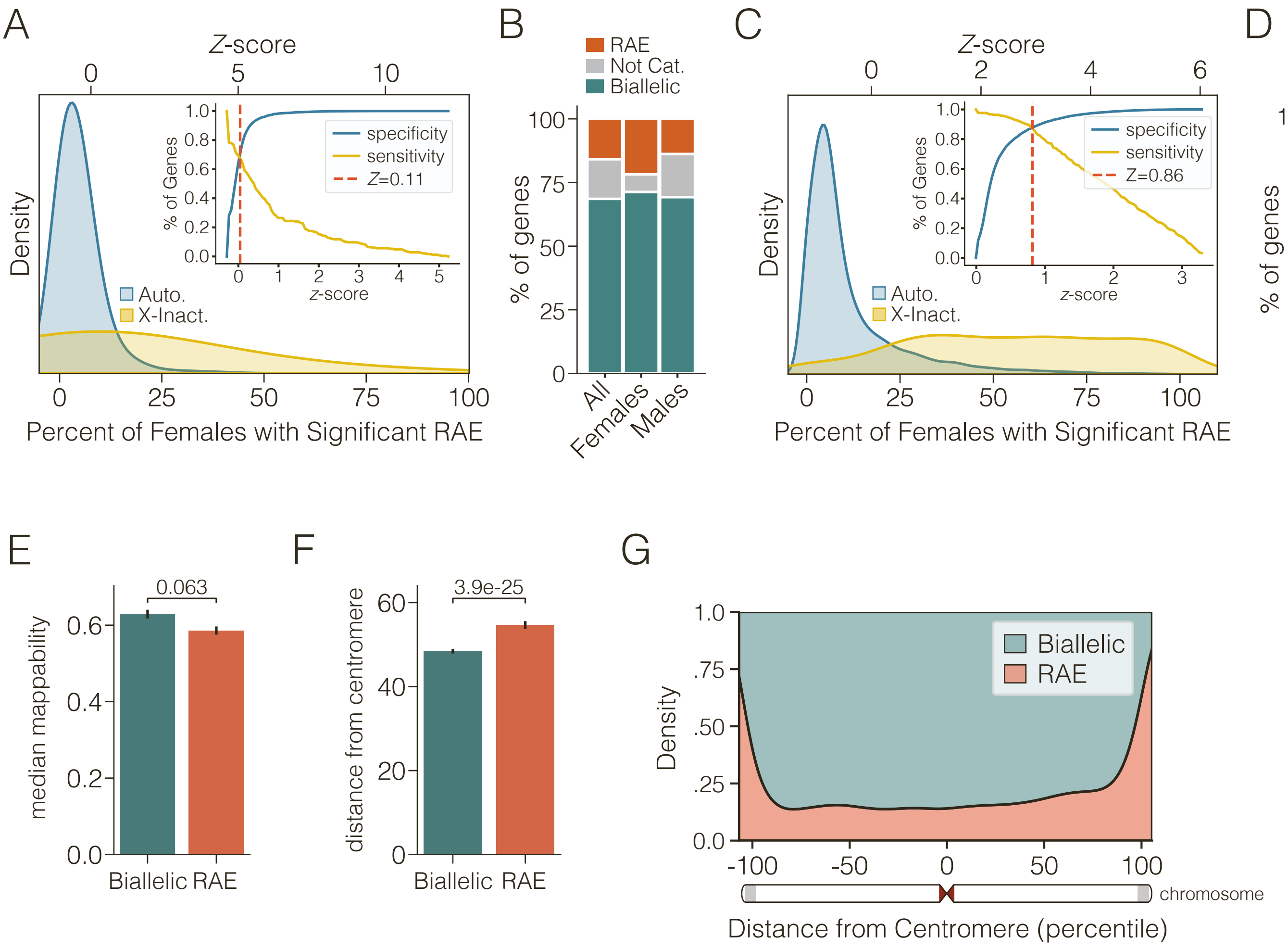
A
Z-score
0
5
10
Density
% of Genes
1.0
0.8
0.6
0.4
0.2
0.0
specificity
sensitivity
Z=0.11
0
1
2
3
4
5
z-score
Auto.
X-Inact.
0
25
50
75
100
Percent of Females with Significant RAE
B
RAE
Not Cat.
Biallelic
% of genes
100
75
50
25
0
All
Females
Males
C
Z-score
0
2
4
6
Density
% of Genes
1.0
0.8
0.6
0.4
0.2
0.0
specificity
sensitivity
Z=0.86
0
1
2
3
z-score
Auto.
X-Inact.
0
25
50
75
100
Percent of Females with Significant RAE
D
RAE
Not Cat.
Biallelic
% of genes
100
75
50
25
0
All
Females
Males
E
median mappability
0.063
0.6
0.4
0.2
0.0
Biallelic
RAE
F
distance from centromere
3.9e-25
60
40
20
0
Biallelic
RAE
G
Density
Biallelic
RAE
1.0
.75
.50
.25
0.0
-100
-50
0
50
100
chromosome
Distance from Centromere (percentile)

Figure S2. The proportion of individuals with putative RAE for each gene in each of the following datasets. Related to Figure 3.

(A) Body Tissues (3-30 tissues); the proportion of GTEx females with putative RAE (FDR 10%) for autosomal and XCI genes, plotted as kernel densities. The top x-axis shows corresponding *Z*-scores. Inset: The sensitivity and specificity of distinguishing XCI vs. autosomal genes with putative RAE at various *Z*-score cut-offs. The dashed red line shows the optimal cut-off deduced (Z=0.86). (B) The proportion of genes identified as biallelic ($Z<=0$), RAE ($Z>=0.86$), or not categorized ($0<Z<0.86$) using all GTEx females or males only using body tissues only. (C) Brain Tissues (3-13 tissues); the proportion of GTEx females with putative RAE (FDR 10%) for autosomal and XCI genes, plotted as kernel densities. The top x-axis shows corresponding *Z*-scores. Inset: The sensitivity and specificity of distinguishing XCI vs. autosomal genes with putative RAE at various *Z*-score cut-offs. The dashed red line shows the optimal cut-off deduced (Z=0.11). (D) Proportion of genes identified as biallelic ($Z<=0$), RAE ($Z>=0.11$), or not categorized ($0<Z<0.11$) using all GTEx females or males only using body tissues only (3-13 tissues). (E) Median per-bp mappability scores for hc-RAE and hc-Biallelic genes in hc-RAE enriched TADs located at telomeres (Kruskal-Wallis ANOVA; $p=0.063$). (F) Distance (as percentiles) from the centromere for hc-biallelic and hc-RAE genes across autosomes. The centromere is located at 0. (Kruskal-Wallis ANOVA; $p=3.86x10^{-25}$). (G) Distance (as percentiles) from the centromere for hc-biallelic (teal) and hc-RAE (orange) genes across all autosomes, plotted as stacked densities. Barplots in (E) and (F) show the standard error of the mean.

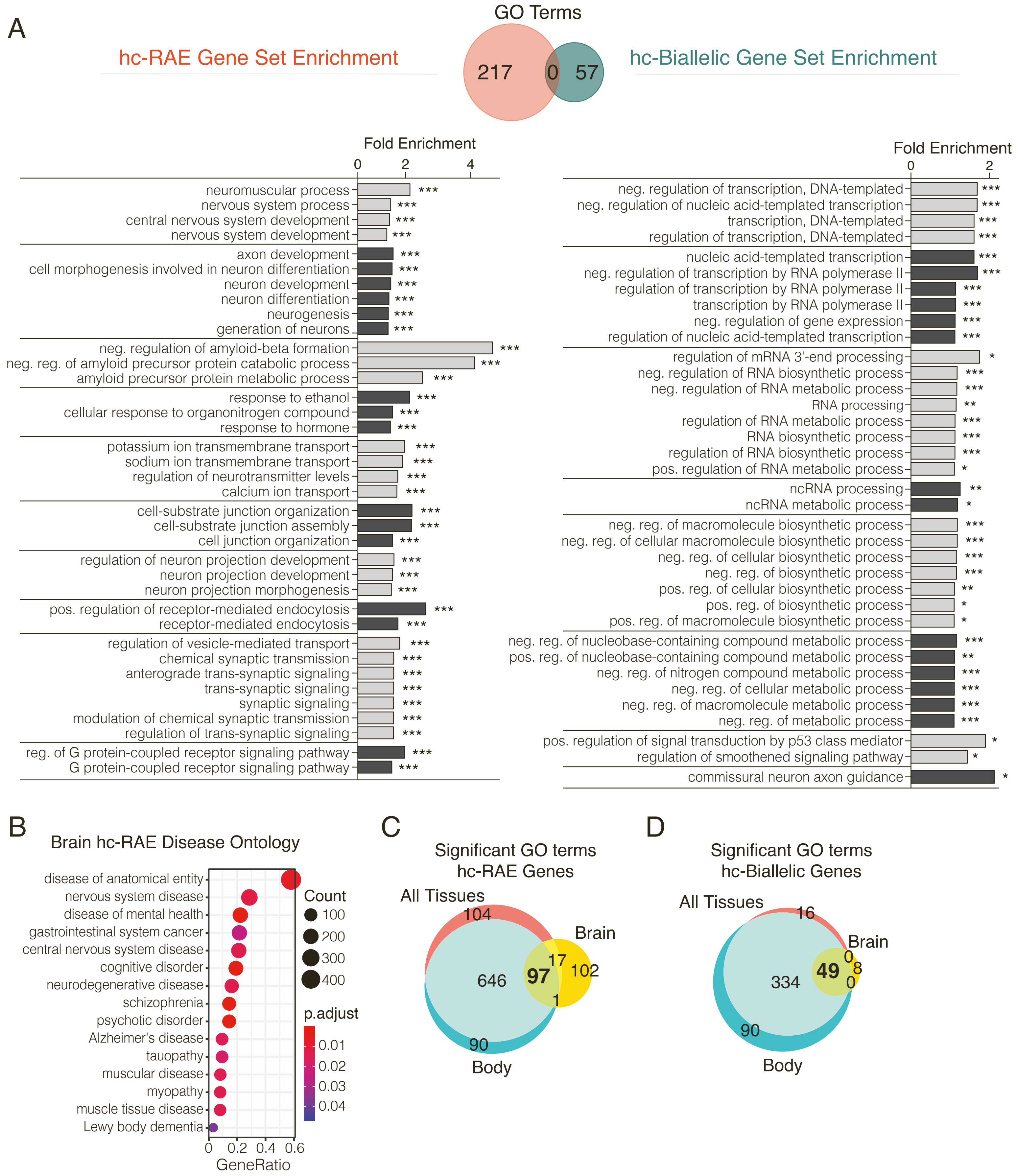
A
GO Terms
hc-RAE Gene Set Enrichment
217
0
57
hc-Biallelic Gene Set Enrichment
Fold Enrichment
0 2 4
neuromuscular process ***
nervous system process ***
central nervous system development ***
nervous system development ***
axon development ***
cell morphogenesis involved in neuron differentiation ***
neuron development ***
neuron differentiation ***
neurogenesis ***
generation of neurons ***
neg. regulation of amyloid-beta formation ***
neg. reg. of amyloid precursor protein catabolic process ***
amyloid precursor protein metabolic process ***
response to ethanol ***
cellular response to organonitrogen compound ***
response to hormone ***
potassium ion transmembrane transport ***
sodium ion transmembrane transport ***
regulation of neurotransmitter levels ***
calcium ion transport ***
cell-substrate junction organization ***
cell-substrate junction assembly ***
cell junction organization ***
regulation of neuron projection development ***
neuron projection development ***
neuron projection morphogenesis ***
pos. regulation of receptor-mediated endocytosis ***
receptor-mediated endocytosis ***
regulation of vesicle-mediated transport ***
chemical synaptic transmission ***
anterograde trans-synaptic signaling ***
trans-synaptic signaling ***
synaptic signaling ***
modulation of chemical synaptic transmission ***
regulation of trans-synaptic signaling ***
reg. of G protein-coupled receptor signaling pathway ***
G protein-coupled receptor signaling pathway ***
Fold Enrichment
0 2
neg. regulation of transcription, DNA-templated ***
neg. regulation of nucleic acid-templated transcription ***
transcription, DNA-templated ***
regulation of transcription, DNA-templated ***
nucleic acid-templated transcription ***
neg. regulation of transcription by RNA polymerase II ***
regulation of transcription by RNA polymerase II ***
transcription by RNA polymerase II ***
neg. regulation of gene expression ***
regulation of nucleic acid-templated transcription ***
regulation of mRNA 3'-end processing *
neg. regulation of RNA biosynthetic process ***
neg. regulation of RNA metabolic process ***
RNA processing **
regulation of RNA metabolic process ***
RNA biosynthetic process ***
regulation of RNA biosynthetic process ***
pos. regulation of RNA metabolic process *
ncRNA processing **
ncRNA metabolic process *
neg. reg. of macromolecule biosynthetic process ***
neg. reg. of cellular macromolecule biosynthetic process ***
neg. reg. of cellular biosynthetic process ***
neg. reg. of biosynthetic process ***
pos. reg. of cellular biosynthetic process **
pos. reg. of biosynthetic process *
pos. reg. of macromolecule biosynthetic process *
neg. reg. of nucleobase-containing compound metabolic process ***
pos. reg. of nucleobase-containing compound metabolic process **
neg. reg. of nitrogen compound metabolic process ***
neg. reg. of cellular metabolic process ***
neg. reg. of macromolecule metabolic process ***
neg. reg. of metabolic process ***
pos. regulation of signal transduction by p53 class mediator *
regulation of smoothened signaling pathway *
commissural neuron axon guidance *
B
Brain hc-RAE Disease Ontology
disease of anatomical entity
nervous system disease
disease of mental health
gastrointestinal system cancer
central nervous system disease
cognitive disorder
neurodegenerative disease
schizophrenia
psychotic disorder
Alzheimer's disease
tauopathy
muscular disease
myopathy
muscle tissue disease
Lewy body dementia
0 0.2 0.4 0.6
GeneRatio
Count
100
200
300
400
p.adjust
0.01
0.02
0.03
0.04
C
Significant GO terms
hc-RAE Genes
All Tissues
104
Brain
646
97
17
102
1
90
Body
D
Significant GO terms
hc-Biallelic Genes
All Tissues
16
Brain
334
49
0
8
0
90
Body

Figure S3. Biological Functions of hc-RAE and hc-Biallelic genes in the Brain. Related to Figure 4.

(A) Fold Enrichments for a subset of significant Gene Ontology (GO) terms for hc-RAE (left) and hc-Biallelic (right) gene sets from brain tissues. Venn diagram shows there is no overlap of significant GO terms for either gene set. See **Table S4** for full GO results. (B-H adjusted p-values, FDR 10%; *p<.01, **p<.001, ***p<.0001) (B) Top 15 significant Disease Ontology (DO) terms for hc-RAE genes identified in brain tissues. See **Table S5** for full DO results. (C) Venn diagram depicting the intersection of significant GO terms for hc-RAE genes using all tissues, brain, and body only. 97 GO terms are shared between all three datasets. (D) Venn diagram depicting the intersection of significant GO terms for hc-biallelic genes using all tissues, brain, and body only. 49 GO terms are shared between all three datasets.

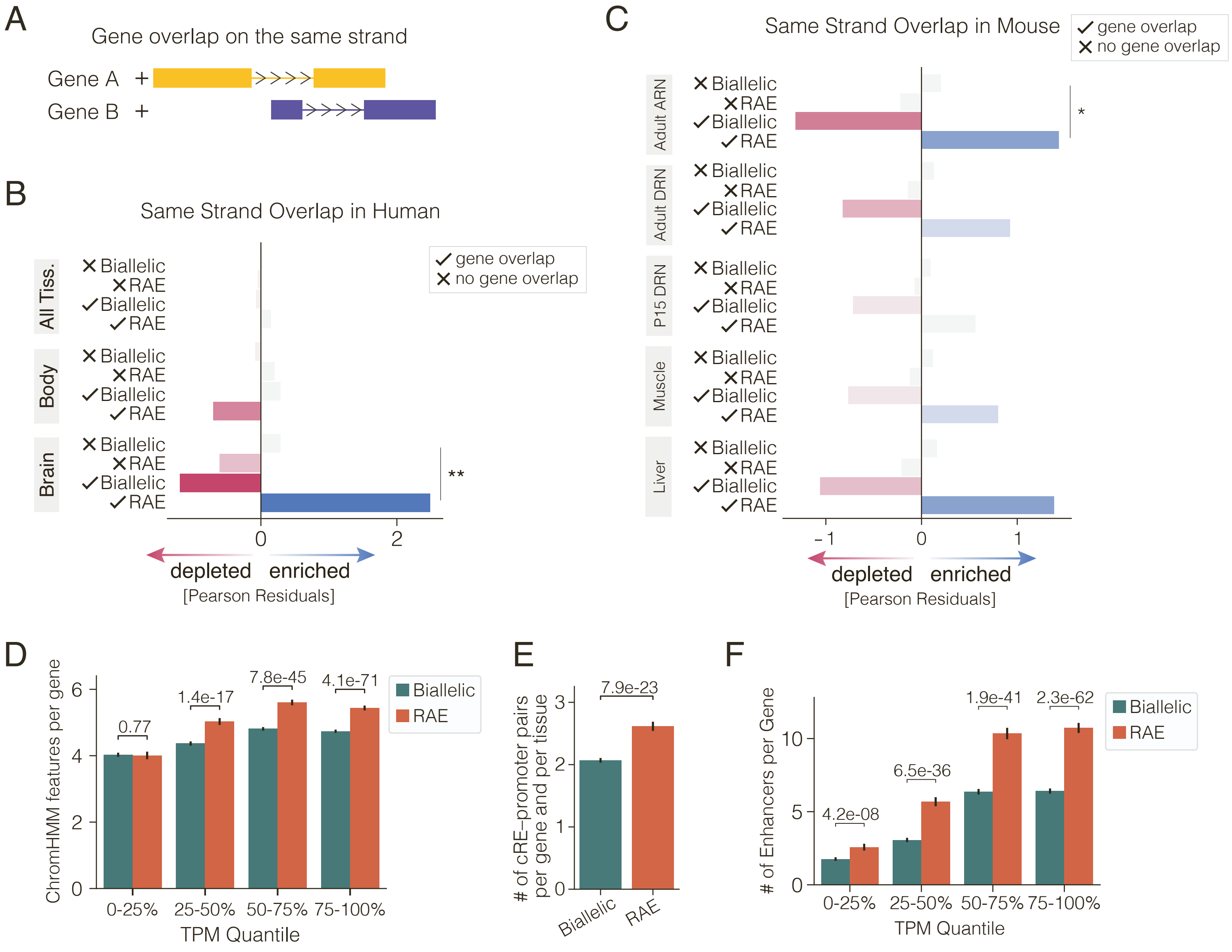
A
Gene overlap on the same strand
Gene A +
Gene B +
B
Same Strand Overlap in Human
gene overlap
no gene overlap
All Tiss.
Body
Brain
Biallelic
RAE
**
0
2
depleted
enriched
[Pearson Residuals]
C
Same Strand Overlap in Mouse
gene overlap
no gene overlap
Adult ARN
Adult DRN
P15 DRN
Muscle
Liver
Biallelic
RAE
*
-1
0
1
depleted
enriched
[Pearson Residuals]
D
ChromHMM features per gene
0.77
1.4e-17
7.8e-45
4.1e-71
Biallelic
RAE
0-25%
25-50%
50-75%
75-100%
TPM Quantile
E
# of cRE–promoter pairs per gene and per tissue
7.9e-23
Biallelic
RAE
F
# of Enhancers per Gene
4.2e-08
6.5e-36
1.9e-41
2.3e-62
Biallelic
RAE
0-25%
25-50%
50-75%
75-100%
TPM Quantile

Figure S4. Mechanisms regulating differential allelic expression. Related to Figure 5.

(A) Schematic of same strand (+/+ or -/-) gene overlap. (B) Pearson residuals for same strand gene overlap in hc-RAE vs. hc-Biallelic gene sets for all, body, or brain tissues (Pearson Chi-Square Test; all-tissues, $p=.86$; body, $p=.43$; brain, $p=4.49x10^{-03}$). (C) Pearson residuals for same strand gene overlap in RAE and Biallelic genes in P15 and adult mouse brain and body (Huang and Ferris, 2017). (Pearson Chi-Square Test; adult ARN, $p=0.049$). See **Table S6** for full data. (D) Number of different ChromHMM features (6 total, see Methods) for RAE and Biallelic genes binned by expression (TPM) quartiles. RAE genes have a higher diversity of intragenic cCREs than biallelic genes at all expression levels, except for the lowest (Kruskal-Wallis ANOVA; 1st quartile, $p=0.77$; 2nd, $p=1.4x10^{-17}$; 3rd, $p=7.8x10^{-45}$; 4th, $p=4.1x10^{-71}$) (E) Number of promoter-cRE pairs for cREs contacting Biallelic gene promoters vs. RAE gene promoters, on average per tissue (one-way ANOVA, $p=7.9x10^{-23}$). (F) Number of GeneHancer enhancers regulating RAE and Biallelic genes binned by expression (TPM) quartiles. RAE genes have a higher number of enhancers than biallelic genes at all expression levels (Kruskal-Wallis ANOVA; 1st quartile, $p=4.2x10^{-08}$; 2nd, $p=6.5x10^{-36}$; 3rd, $p=1.9x10^{-41}$; 4th, $p=2.3x10^{-62}$). Error Bars for (D), (E), (F), show standard error of the mean.
